# PER2 integrates circadian disruption and pituitary tumorigenesis

**DOI:** 10.7150/thno.82995

**Published:** 2023-04-29

**Authors:** Lianxia Guo, Haobin Cen, Jiaxian Weng, Yiting He, Xiaocao Guo, Di He, Kaisheng Liu, Shuyi Duan, Jing Yang, Xiaojian Zhang, Zifei Qin, Yong Wan, Zhiyong Chen, Baojian Wu

**Affiliations:** 1 Institute of Molecular Rhythm and Metabolism, Guangzhou University of Chinese Medicine, Guangzhou, China.; 2 Department of Public Health and Preventive Medicine, School of Medicine, Jinan University, Guangzhou, China.; 3 Guangdong Provincial Clinical Research Center for Geriatrics, Shenzhen Clinical Research Center for Geriatrics, Shenzhen People's Hospital (The Second Clinical Medical College, Jinan University; The First Affiliated Hospital, Southern University of Science and Technology), Shenzhen, Guangdong, China.; 4 Department of Pharmacy, the First Affiliated Hospital of Zhengzhou University, Zhengzhou 450052, China.; 5 Department of Neurosurgery, the First Affiliated Hospital of Jinan University, Guangzhou, China.; 6 Minimally Invasive Treatment Center for Pituitary Adenoma of Jinan University, Guangzhou, China.

**Keywords:** Pituitary adenoma, Circadian disruption, PER2, Cell cycle, Apoptosis.

## Abstract

**Rationale:** The role of circadian clock in pituitary tumorigenesis remains elusive. Here we investigate whether and how circadian clock modulates the development of pituitary adenomas.

**Methods and Results:** We found altered expression of pituitary clock genes in patients with pituitary adenomas. In particular, *PER2* is prominently upregulated. Further, jetlagged mice with PER2 upregulation have accelerated growth of GH3 xenograft tumor. Conversely, loss of *Per2* protects mice against developing estrogen-induced pituitary adenoma. Similar antitumor effect is observed for SR8278, a chemical that can decrease pituitary PER2 expression. RNA-seq analysis suggests involvement of cell cycle disturbance in PER2 regulation of pituitary adenoma. Subsequent *in vivo* and cell-based experiments validate that PER2 induces pituitary expression of *Ccnb2*, *Cdc20* and *Espl1* (three cell cycle genes) to facilitate cell cycle progression and inhibit apoptosis, thereby promoting pituitary tumorigenesis. Mechanistically, PER2 regulates the transcription of *Ccnb2*, *Cdc20* and *Espl1* through enhancing the transcriptional activity of HIF-1α. HIF-1α trans-activates *Ccnb2*, *Cdc20* and *Espl1* via direct binding to its specific response element in the gene promoters.

**Conclusion:** PER2 integrates circadian disruption and pituitary tumorigenesis. These findings advance our understanding of crosstalk between circadian clock and pituitary adenomas and highlight the relevance of clock-based approaches in disease management.

## Introduction

Pituitary adenomas, monoclonal in nature, account for 15-25% of intracranial neoplasms and are associated with significant morbidity such as headaches and visual disturbances due to local mass effects 1. When pituitary adenomas hypersecrete hormones, they can lead to severe clinical syndromes, which impinge on various organ systems and can be lethal 2. For instance, growth hormone (GH)-secreting pituitary adenoma (GHPA, also known as somatotropinoma) causes gigantism and acromegaly 3. Prolactin (PRL)-secreting pituitary adenoma (PRLPA, also known as prolactinoma) can give rise to hypogonadism and galactorrhea 4. The exact causes of pituitary adenomas are unknown and the disease pathogenesis is poorly understood. It is believed that disrupted cell cycle regulation and aberrant growth factor signaling are involved in pituitary tumorigenesis 5. Effective drugs are unavailable or lacking for management of pituitary adenomas 5. Although drugs such as somatostatin analogues and dopamine agonists are approved for treating GHPA and PRLPA, about 20% of patients are unresponsive to these medicines or intolerant of their adverse effects 6. Pituitary adenomas can be usually treated by transsphenoidal surgery. However, surgical removal of pituitary tumor is associated with various post-operative morbidity such as diabetes insipidus, rhinorrhea and epistaxis 7. Therefore, it is of clinical relevance to understand the molecular events and mechanisms underlying pituitary tumorigenesis and to develop novel targeted therapies.

Circadian clock is an intrinsic timing system which generates around 24-h rhythmicity in biochemical, physiological and behavioral processes, allowing living organisms to anticipate daily changes in the environment (e.g., the light-dark cycle, temperature, and humidity) imposed by the Earth's rotation 8. In mammals, molecular clock is present in virtually every cell, and functions in a self-sustained (auto-regulatory) manner relying on several interlocked transcriptional-translational negative feedback loops 9. The clock genes such as *Bmal1* (brain and muscle Arnt-like protein 1), *Clock* (circadian locomotor output cycles kaput), *Per* (period), *Cry* (cryptochrome), *Ror* (retinoic acid-related orphan receptor), *Nr1d1* (nuclear receptor subfamily 1 group D member 1), *Dbp* (D site-binding protein) and *Nfil3* (nuclear factor interleukin-3), and their proteins are key participants in the core and auxiliary feedback loops 10. In the core loop, BMAL1 associates with CLOCK to form a heterodimer that activates the transcription of *Per* and *Cry* (as well as many other target genes), whose proteins feedback and oppose the action of BMAL1/CLOCK to inhibit target gene transcription 11. Clocks in body tissues are deemed to be hierarchal: the suprachiasmatic nucleus (SCN) of brain harbors a central clock which coordinates the clocks in other parts of the brain and in peripheral tissues such as the liver, lung and kidney 12.

Mammalian cell proliferation and apoptosis are closely linked to the clock system. Many genes involved in cell cycle and apoptosis [ e.g., *c-Myc* (cellular myelocytomatosis oncogene), *Ccnd1* (cyclin-D1), *Wee1* [WEE 1 homolog 1 (S. pombe)]*, Bcl2* (B-cell lymphoma 2), *Bid* (BH3-interacting domain death agonist) and *Bak1* (BRI1-associated kinase 1)] oscillate robustly in a circadian manner and are under the control of circadian clocks 13,14. Perturbations of circadian rhythms (e.g., due to shift work, sleep disruption, mistimed feeding and chronic jet lag) in humans are associated with an increased risk for tumor formation and developing most common types of carcinomas such as prostate, breast, colorectal, lung, and skin cancers 15. Likewise, genetic and physiologic disruptions of circadian rhythms predispose experimental animals to tumor growth and carcinogenesis 16. Accordingly, night shift work (pertinent to circadian disruption) is classified as “probably carcinogenic to humans” (Group 2A) by the IARC (International Agency for Research on Cancer) 17. A number of studies have further uncovered inhibitory effects of core clock components (such as *Bmal1* and *Clock*) on tumor proliferation and growth in various cancer types, including hepatocellular carcinoma, nasopharyngeal carcinoma, tongue squamous cell carcinoma, colon, pancreatic and breast cancers 18. Their anti-cancer mechanisms involve the promotion of oncoprotein [e.g., c-MYC, E2F (E2 promoter binding factor) and TLK2 (tousled like kinase 2)] degradation, cell cycle arrest, apoptosis, cytotoxic immunity and metabolic defects 19. Notably, the roles of clock components in tumorigenesis depend on cancer cell type or status, as they (e.g., *Bmal1, Clock* and *Cry1*) exert tumor-promoting effects for some cell types such as mesothelioma, glioblastoma stem cells and leukemia stem cells 20. Tight relationships between circadian rhythms and cancer pathogenesis have promoted extensive research and development of chronotherapeutic approaches for cancer therapy 21.

Reportedly, patients with pituitary adenomas are associated with severe disturbances in circadian rhythms, as exemplified by disrupted sleep-wake cycle, altered diurnal patterns of melatonin and cortisol, and disturbed movement rhythm 22,23. These observations suggest potential involvement of circadian rhythms in pituitary tumorigenesis. However, it remains unknown whether and how circadian clock indeed contributes to the pathogenesis of pituitary adenomas. Here, we aimed to investigate the role of circadian clock in pituitary tumorigenesis. We centered on GHPA and PRLPA as they are two most common types of pituitary adenomas. Of clock components PER2 was up-regulated in the tumors in patients with GHPA and PRLPA and in a mouse model. Loss of *Per2* in mice decreased the growth rate of transplanted GH3 tumor, and protected from estrogen-induced PRLPA. *In vivo* and *in vitro* experiments validated that inhibition of *Per2* was able to repress pituitary cell proliferation. Mechanistically, PER2 promoted pituitary cell proliferation through inducing cell cycle genes [*Ccnb2* (cyclin-B2), *Cdc20* (cell division cycle 20) and *Espl1* (extra spindle pole bodies like 1)]. Induction of cell cycle genes by PER2 was attained via enhancement of HIF-1α (hypoxia-inducible factor 1α) -mediated transactivation. These findings establish PER2 as an integrator of circadian clock with pituitary adenomas, providing new insights to the molecular mechanisms behind clock-controlled tumorigenesis.

## Results

### Disruption of pituitary clock genes in patients with pituitary adenomas

**Figure 1:**
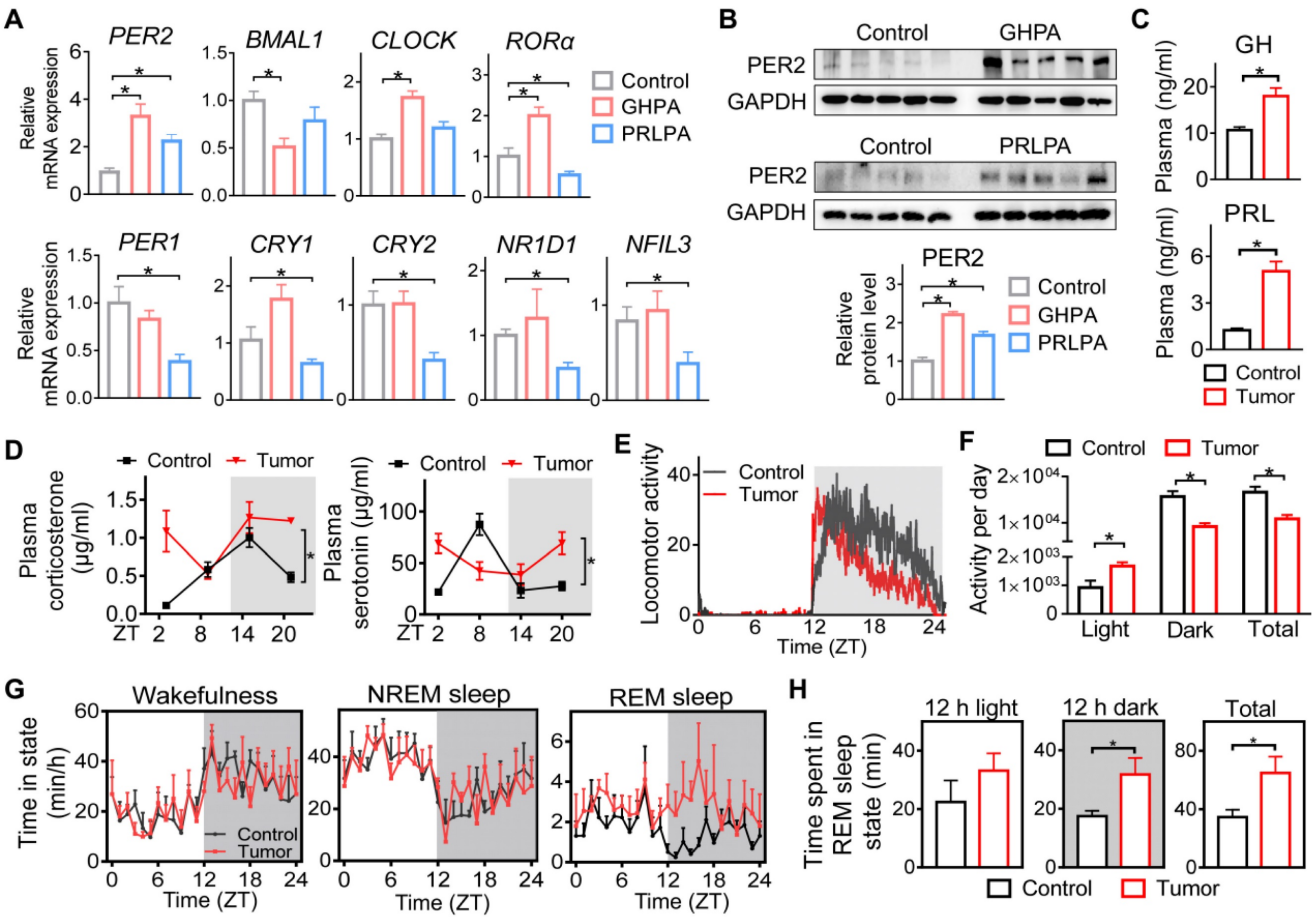
**Disruption of pituitary clock in patients and mice with pituitary adenomas.** (A) Pituitary mRNA expression of core clock genes in patients with GHPA and PRLPA. (B) Pituitary PER2 protein expression in patients with GHPA and PRLPA. Please note that two of ten pituitary samples from ten individuals in each group were pooled for western blotting. (C) Plasma GH and PRL levels in GH3 xenograft tumor-bearing and control mice. (D) Diurnal patterns of plasma corticosterone and serotonin in tumor-bearing and control mice. (E) Wheel-running activities recorded at a 1 min interval for GH3 xenograft tumor-bearing and control mice under a 12 h light/dark cycle. (F) Wheel-running activities in the light and dark phases and total daily wheel-running activities. (G) Daily patterns of wake, NREM, and REM sleep time for tumor-bearing and control mice. (H) A comparison of REM sleep time between the light and dark phases. In panel A, data are mean ± SEM (*n* = 10 biologically independent samples). In panels except A, data are mean ± SEM (*n* = 5 biologically independent samples). In panels A, B, C, F and H, *p < 0.05 (t-test). In panel D, *p < 0.05 (two-way ANOVA).

We collected surgical pituitary samples from patients (at 9:00 AM~12:00 PM) with GHPA or PRLPA. Control pituitary samples were autopsy specimens from individuals who died (between 9:00 AM and 12:00 PM) from accidental causes. We found that GHPA and PRLPA were associated with altered pituitary expression of core circadian clock genes including *BMAL1*,* CLOCK, PER1, PER2, CRY1, CRY2, RORα, NR1D1* and *NFIL3* (Figure 1A). Notably, *PER2* was consistently up-regulated in both GHPA and PRLPA, and was one of the clock genes altered the most (Figure 1A). Western blotting analysis conformed that pituitary PER2 protein was up-regulated in patients of GHPA and PRLPA (Figure 1B). Likewise, pituitary PER2 was up-regulated in a mouse model of PRLPA induced by estrogen, and in pituitary adenoma GH3 and MMQ cells (Figure S1A-B). We additionally analyzed pituitary expression of clock genes in patients with other types of pituitary adenomas, including thyroid-stimulating hormone (TSH)-secreting, adrenocorticotropic hormone (ACTH)-secreting and nonfunctioning pituitary adenomas (Figure S1C). Intriguingly, we also observed elevations in PER2 in these patients (Figure S1C). These findings suggested involvement of circadian clock in pituitary tumorigenesis. In support of this, patients with pituitary adenomas are associated with severe disturbances in circadian rhythms, as exemplified by disrupted sleep-wake cycle, altered diurnal patterns of melatonin and cortisol, and disturbed movement rhythm 22,24-25,26.

We further examined whether circadian rhythms are affected in mice with a xenograft pituitary adenoma. A GHPA xenograft model was generated by subcutaneous injection of GH3 cells into BALB/c nude mice. GH3 xenograft tumor-bearing mice showed a growth hormone-oversecretion phenotype as evidenced by increased plasma GH and PRL (note that GH3 cells also produce PRL), increased body weight and enlarging organs (e.g., the liver and spleen) (Figure 1C and Figure S1D-F). We observed that the diurnal patterns of plasma corticosterone and serotonin were disrupted in tumor-bearing mice (Figure 1D). Moreover, tumor-bearing mice showed attenuated rhythms in wheel-running activities with an increased activity in the daytime and a reduced activity in the nighttime (Figure 1E-F). In addition, they had an altered pattern of sleep-wake cycle with significantly increased REM (rapid eye movement) sleep time in the dark period (Figuref 1G-H). Altogether, pituitary adenomas (particularly GHPA and PRLPA) are associated with disrupted circadian rhythms in behaviors and altered clock genes in pituitary glands.

### Circadian disturbance promotes pituitary tumorigenesis

**Figure 2:**
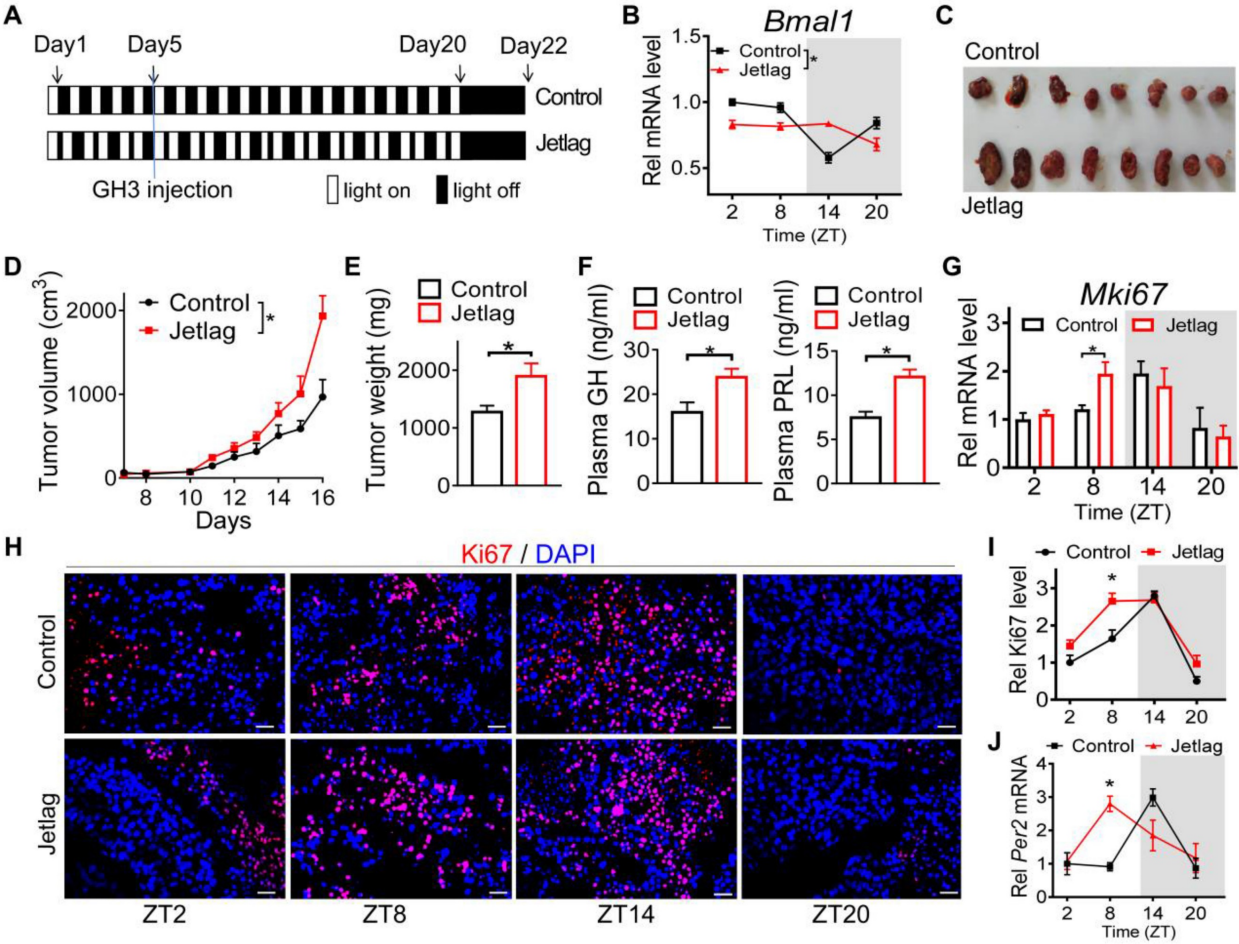
**Circadian disturbance promotes pituitary tumorigenesis.** (A) Experimental scheme for establishment of jetlagged nude mice. (B) Diurnal *Bmal1* mRNA in the tumors of jetlagged and control mice. (C) A photograph of tumors excised from jetlagged and control mice. (D) Tumor growth of jetlagged and control mice. (E) Tumor weight of jetlagged and control mice. (F) Plasma GH and PRL in jetlagged and control mice. (G) Diurnal mRNA expression of *Mki67* in tumors of jetlagged and control mice. (H) Immunofluorescent staining of Ki67 in tumors from jetlagged and control mice (scale bar, 25 µm). (I) Quantitative analysis of Ki-67 staining of tumors from jetlagged and control mice. (J) Diurnal mRNA level of *Per2* in tumors of jetlagged and control mice. Data are mean ± SEM (*n* = 8 biologically independent samples). In panels B, D, G, I and J, *p < 0.05 (two-way ANOVA with Bonferroni post hoc test). In panels E and F, *p < 0.05 (t-test). Rel, relative.

Given that pituitary adenoma is associated with disruption of circadian rhythms, we next tested whether circadian clock has a role in pituitary tumorigenesis. Jetlagged nude mice were established by applying a schedule of 8-h advances in light/dark cycle every 2 days for 20 days, and we initiated subcutaneous injection of GH3 cells in the fifth day (Figure 2A). The mice continued on the light advancing schedule for another 15 days and then were released into constant darkness (Figure 2A). As expected, diurnal *Bmal1* expression was disrupted in the tumor of jetlagged mice (Figure 2B). Intriguingly, jetlagged mice showed an accelerated rate of tumor growth, as evidenced by larger tumor volume and higher tumor mass (Figure 2C-E). They also had higher levels of plasma GH and PRL (Figure 2F). It was noted that the tumor of control mice showed a diurnal oscillation in the proliferation index (Ki67) with a peak level at zeitgeber time (ZT) 14 (early dark phase, Figure 2G-I), paralleling the diurnal pattern of pituitary *Per2* expression (Figure S2). Intriguingly, jetlag increased the proliferation index of GH3 tumor at ZT8 while having no effects at other times of the day (Figure 2G-I). This was associated with up-regulation of *Per2* in GH3 tumor at ZT8 but no changes at other times (Figure 2J). These findings suggested a critical role of circadian clock in pituitary tumorigenesis, and potential involvement of PER2 in circadian regulation of pituitary adenoma.

### *Per2* ablation in mice restrains pituitary tumorigenesis

**Figure 3:**
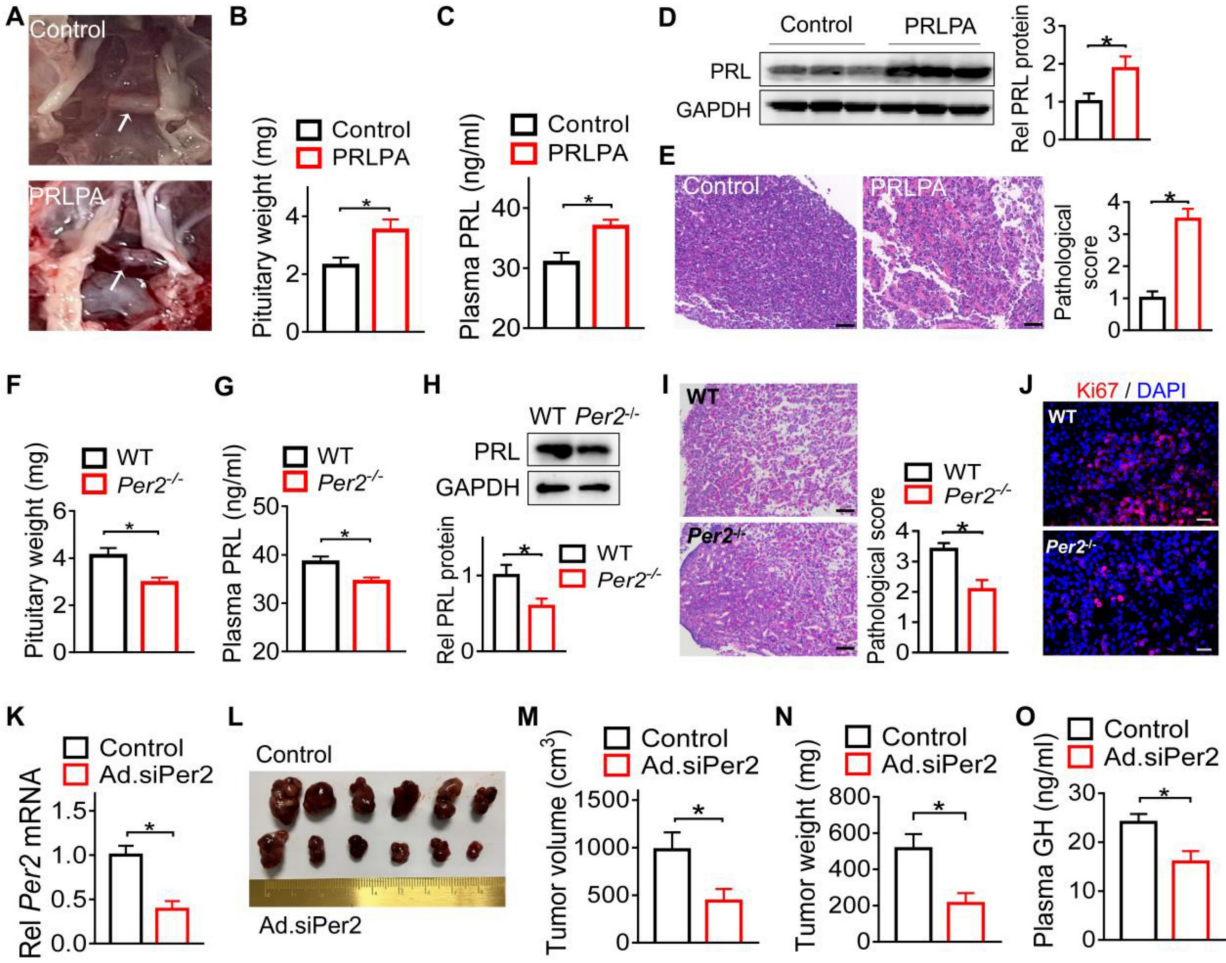
***Per2* ablation in mice restrains pituitary tumorigenesis.** (A) Photographs of pituitary glands from control mice and mice with estrogen-induced PRLPA. (B) Pituitary weight of control mice and mice with estrogen-induced PRLPA. (C) Plasma PRL level in control mice and mice with estrogen-induced PRLPA. (D) Pituitary PRL protein in control mice and mice with estrogen-induced PRLPA. (E) Histopathological examinations of pituitary samples from control mice and mice with estrogen-induced PRLPA (scale bar, 25 µm). (F) Pituitary mass in estrogen-treated* Per2*^-/-^ and wild-type mice. (G) Plasma PRL in estrogen-treated* Per2*^-/-^ and wild-type mice. (H) Pituitary PRL protein in estrogen-treated* Per2*^-/-^ and wild-type mice. (I) Histopathological examinations of pituitary samples from estrogen-treated* Per2*^-/-^ and wild-type mice (scale bar, 25 µm). (J) Ki67 levels in estrogen-treated* Per2*^-/-^ and wild-type mice (scale bar, 25 µm). (K) Knockdown validation for Ad.siPer2 (an adenoviral vector encoding siPer2). (L) A photograph of xenograft tumors from mice injected with Ad.siPer2-treated GH3 cells and control cells. (M) The volume of xenograft tumors from mice injected with Ad.siPer2-treated GH3 cells and control cells. (N) The weight of xenograft tumors from mice injected with Ad.siPer2-treated GH3 cells and control cells. (O) Plasma GH levels in mice injected with Ad.siPer2-treated GH3 cells and control cells. Data are mean ± SEM (*n* = 6 biologically independent samples). *p < 0.05 (t-test). WT, wild-type.

Because among core clock genes, PER2 was prominently disrupted in the pituitary adenomas and *Per2* disruption is related to jetlag-induced GH3 tumor growth (Figures 1 & 2), we next investigated whether PER2 indeed affects pituitary tumorigenesis. Both *Per2*-deficient (*Per2*^-/-^) and wild-type mice were treated with estrogen to induce PRLPA. Estrogen treatment led to PRLPA in wild-type mice as evidenced by hair loss (Figure S3A), enlarged pituitary gland (Figure 3A-B), increased plasma and pituitary PRL (Figure 3C-D), as well as pathological changes (i.e., widespread vacuolation, vascular lakes, nuclear pleomorphism and frequent mitosis, Figure 3E). Interestingly, *Per2* ablation suppressed PRLPA development as evidenced by less hair loss (Figure S3B), lower pituitary mass (Figure 3F), as well as lower plasma and pituitary PRL (Figure 3G-H). This was supported by less extensive pathological changes (Figure 3I) and a lower level of Ki67 (Figure 3J and Figure S3C) in *Per2* knockout mice. We further tested the role of PER2 in development of GH3 xenograft tumor. To this end, we generated *Per2*-deficient GH3 cells by transfection of the cells with an adenoviral vector encoding siPer2 (named Ad.siPer2). We confirmed effective knockdown of *Per2* by Ad.siPer2 in GH3 cells (Figure 3K). Mice were subcutaneously injected with *Per2-*deficient or control GH3 cells to induce a xenograft tumor. We found that deficiency of *Per2* retarded the growth of the GH3 tumor as revealed by reduced tumor volume (Figure 3L-M) and tumor mass (Figure 3N) as well as a lower plasma GH level (Figure 3O). Taken together, *Per2* ablation has an inhibitory effect on pituitary tumorigenesis, implicating PER2 as a pituitary tumor-promoting factor.

### Loss of *Per2* suppresses pituitary cell cycle progression in mice

**Figure 4:**
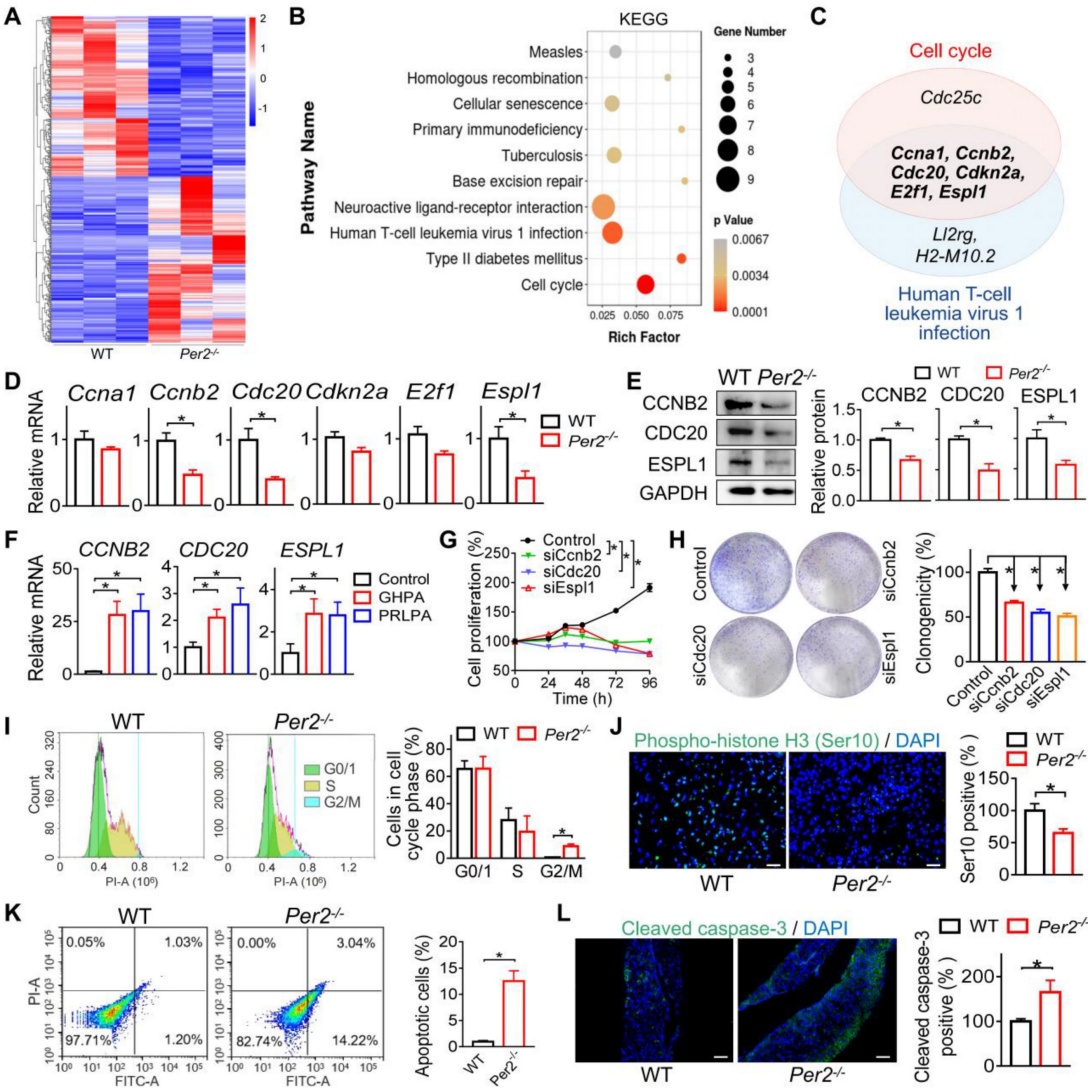
** Loss of *Per2* suppresses pituitary cell cycle progression in mice.** (A) Heatmap for differentially expressed genes (DEGs) in pituitary glands from estrogen-treated *Per2*^-/-^ and wild-type mice. Red indicates high expression, and blue indicates low expression of genes. (B) KEGG enrichment analysis of DEGs between *Per2*^-/-^ and control mice. (C) Venn diagram showing six cell cycle genes (i.e., *Ccna1*, *Ccnb2*, *Cdc20*, *Cdkn2a*, *E2f1*, and *Espl1*) are commonly found in cell cycle and HTLV-1 infection pathways. (D) Pituitary mRNAs of *Ccnb2, Cdc20, Espl1, Ccna1, Cdkn2a* and *E2f1* in *Per2*^-/-^ and control mice. (E) Pituitary proteins of CCNB2, CDC20 and ESPL1 in *Per2*^-/-^ and control mice. (F) Patients with GHPA and PRLPA are associated with increased levels of *CCNB2*,* ESPL1* and *CDC20.* (G) Gene silencing of *Ccnb2, Cdc20 or Espl1* leads to reduced viability of GH3 cells. (H) Gene silencing of *Ccnb2, Cdc20 or Espl1* leads to reduced colony formation of GH3 cells. (I) Loss of *Per2* in mice arrests pituitary cells at G2/M phase. (J) Fluorescent staining against phospho-histone H3 (Ser 10), showing that loss of *Per2* in mice reduces the number of mitotic pituitary cells (scale bar, 25 µm). (K) Flow cytometry results, showing that *Per2* knockout promotes the apoptosis of pituitary cells in mice. (L) Fluorescent staining against cleaved caspase-3, showing that *Per2* knockout promotes the apoptosis of pituitary cells in mice (scale bar, 50 µm). In panels A, D, E, G, H, I, J, K and L, data are mean ± SEM (*n* = 3 biologically independent samples). In panel F, data are mean ± SEM (*n* = 10 biologically independent samples). In panels D, E, F, H, I, J, K and L, *p < 0.05 (t-test). In panel G, *p < 0.05 (two-way ANOVA ). WT, wild-type.

To explore the mechanisms by which PER2 regulates pituitary tumorigenesis, we performed transcriptomic analyses using pituitary glands from estrogen-treated *Per2*^-/-^ and control mice at ZT14 (when *Per2* has the largest different expression between two genotypes) (Figure S2A). A total of 425 differentially expressed genes (DEGs) were identified in pituitary glands between the two genotypes (Figure 4A). Based on KEGG analyses, these genes were enriched in several pathways such as cell cycle and human T-cell leukemia virus 1 (HTLV-1) infection (Figure 4B and Table S1). Notably, six cell cycle genes [i.e., *Ccna1* (cyclin A1), *Ccnb2*, *Cdc20*, *Cdkn2a* (cyclin dependent kinase inhibitor 2A), *E2f1* (E2 promoter binding factor 1), and* Espl1*] were commonly found in enriched pathways of both cell cycle and HTLV-1 infection (Figure 4C), suggesting involvement of cell cycle disturbance in PER2 regulation of pituitary adenoma. It was also noted that cell cycle was one of enriched terms according to Gene Ontology (GO) analyses (Figure S4). RT-qPCR assays showed that loss of *Per2* indeed decreased the mRNAs of pituitary *Ccnb2*,* Cdc20* and *Espl1*, while having minimal effects on *Ccna1*,* Cdkn2a* and* E2f1* mRNAs (Figure 4D). Consistently, pituitary proteins of CCNB2, CDC20 and ESPL1 were significantly reduced in the knockout mice (Figure 4E). Indeed, patients with GHPA and PRLPA were associated with increased levels of *CCNB2*,* CDC20* and *ESPL1* (Figure 4F). Gene silencing of each of the three genes led to reduced cell viability and colony formation of GH3 cells, confirming their roles in pituitary cell proliferation (Figure 4G-H). We thus further examined the role of cell cycle in PER2 regulation of pituitary tumorigenesis. We found that loss of *Per2* in mice arrested pituitary cells at G2/M phase (Figure 4I). In addition, fluorescent staining against S10-phosphorylated histone H3, a marker for M-phase, showed that loss of *Per2* in mice reduced the number of mitotic cells (Figure 4J). Moreover, *Per2* knockout promoted the apoptosis of pituitary cells in mice according to the flow cytometry and cleaved caspase-3 staining assays (Figure 4K-L). These results indicated that PER2 regulates pituitary tumorigenesis by promoting cell cycle progression and inhibiting apoptosis.

### PER2 regulates cell cycle progression in GH3 and MMQ cells

**Figure 5:**
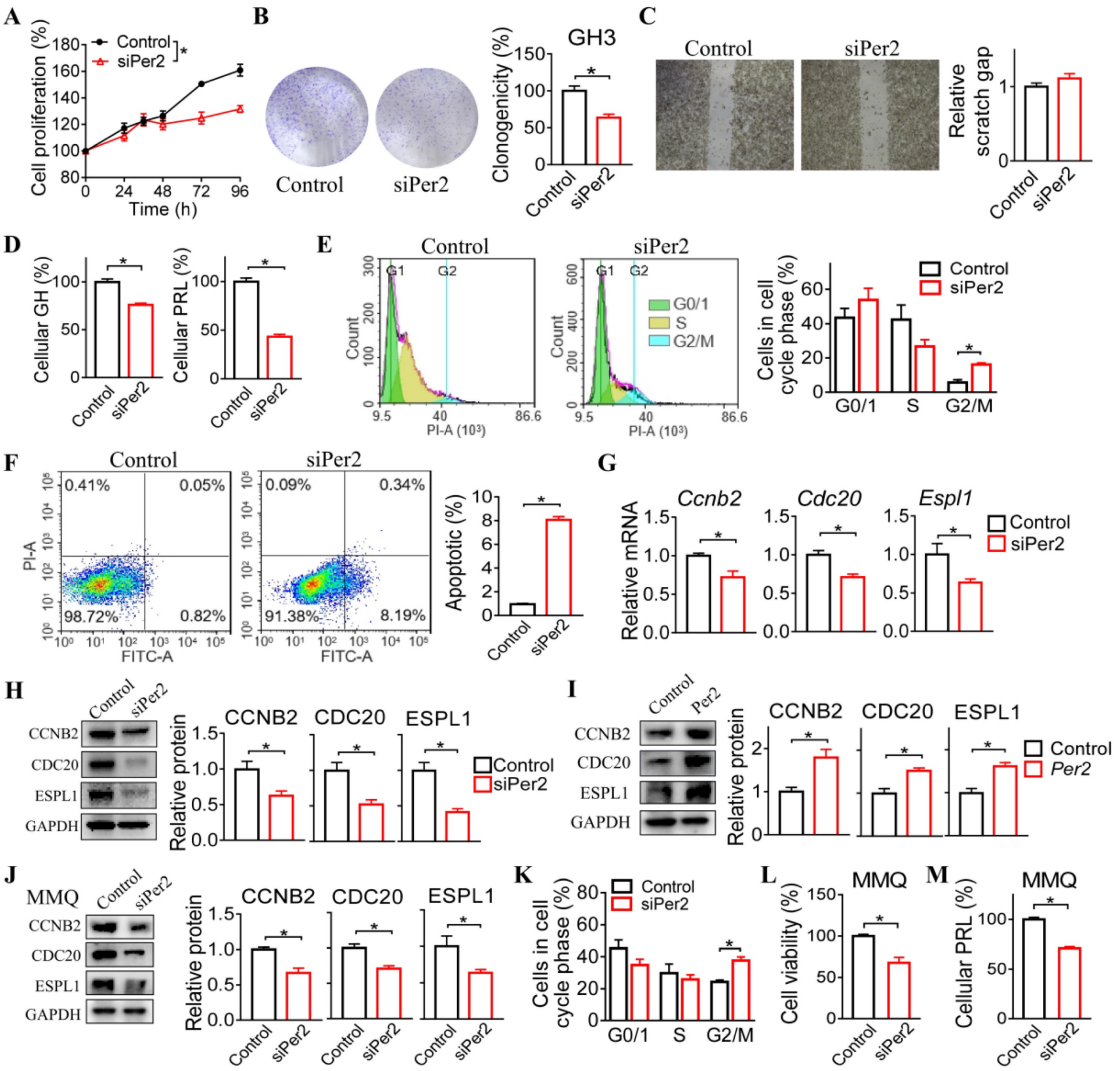
** PER2 regulates cell cycle progression in GH3 and MMQ cells.** (A) Effects of *Per2* knockdown on viability of GH3 cells. (B) Effects of *Per2* knockdown on colony formation of GH3 cells (C) Effects of *Per2* knockdown on migration ability of GH3 cells. (D) Effects of *Per2* knockdown on GH and PRL levels in GH3 cells. (E) Effects of *Per2* knockdown on cell cycle phase distribution of GH3 cells. (F) Effects of *Per2* knockdown on apoptosis of GH3 cells. (G) Effects of *Per2* knockdown on mRNA expression of *Ccnb2, Cdc20* and* Espl1* in GH3 cells. (H) Effects of *Per2* knockdown on protein levels of CCNB2, CDC20 and ESPL1 in GH3 cells. (I) Effects of *Per2* overexpression on protein levels of CCNB2, CDC20 and ESPL1 in GH3 cells. (J) Effects of *Per2* knockdown on mRNA levels of *Ccnb2, Cdc20* and* Espl1* in MMQ cells. (K) Effects of *Per2* knockdown on cell cycle phase distribution of MMQ cells. (L) Effects of *Per2* knockdown on viability of MMQ cells. (O) Effects of *Per2* knockdown on PRL level in MMQ cells. In panel A, data are mean ± SEM (*n* = 6 biologically independent samples). *p < 0.05 (two-way ANOVA). In all panels except A, data are mean ± SEM (*n* = 3 biologically independent samples). *p < 0.05 (t-test).

We next investigated the regulatory effects of PER2 on cell cycle progression in GH3 and MMQ cells. We observed that *Per2* knockdown reduced the viability and colony formation of GH3 cells (Figure 5A-B). In contrast, the migration ability of GH3 cells was unaffected (Figure 5C). In the meantime, *Per2* knockdown led to reduced cellular GH and PRL levels (Figure 5D). Furthermore, *Per2* silencing arrested GH3 cells at the G2/M phase and thus promoted cell apoptosis (Figure 5E-F). In addition, knockdown of *Per2* decreased both mRNA and protein levels of CCNB2, CDC20 and ESPL1 in GH3 cells (Figure 5G-H). Consistent with this, *Per2* overexpression up-regulated the protein levels of CCNB2, CDC20 and ESPL1 in GH3 cells (Figure 5I). Similar inhibitory effects of *Per2* ablation on cell cycle progression were observed in MMQ cells (Figure 5J-M). Overall, these findings supported a critical role of PER2 in regulation of pituitary cell cycle and tumor growth.

### Inhibition of PER2 limits pituitary tumorigenesis

**Figure 6:**
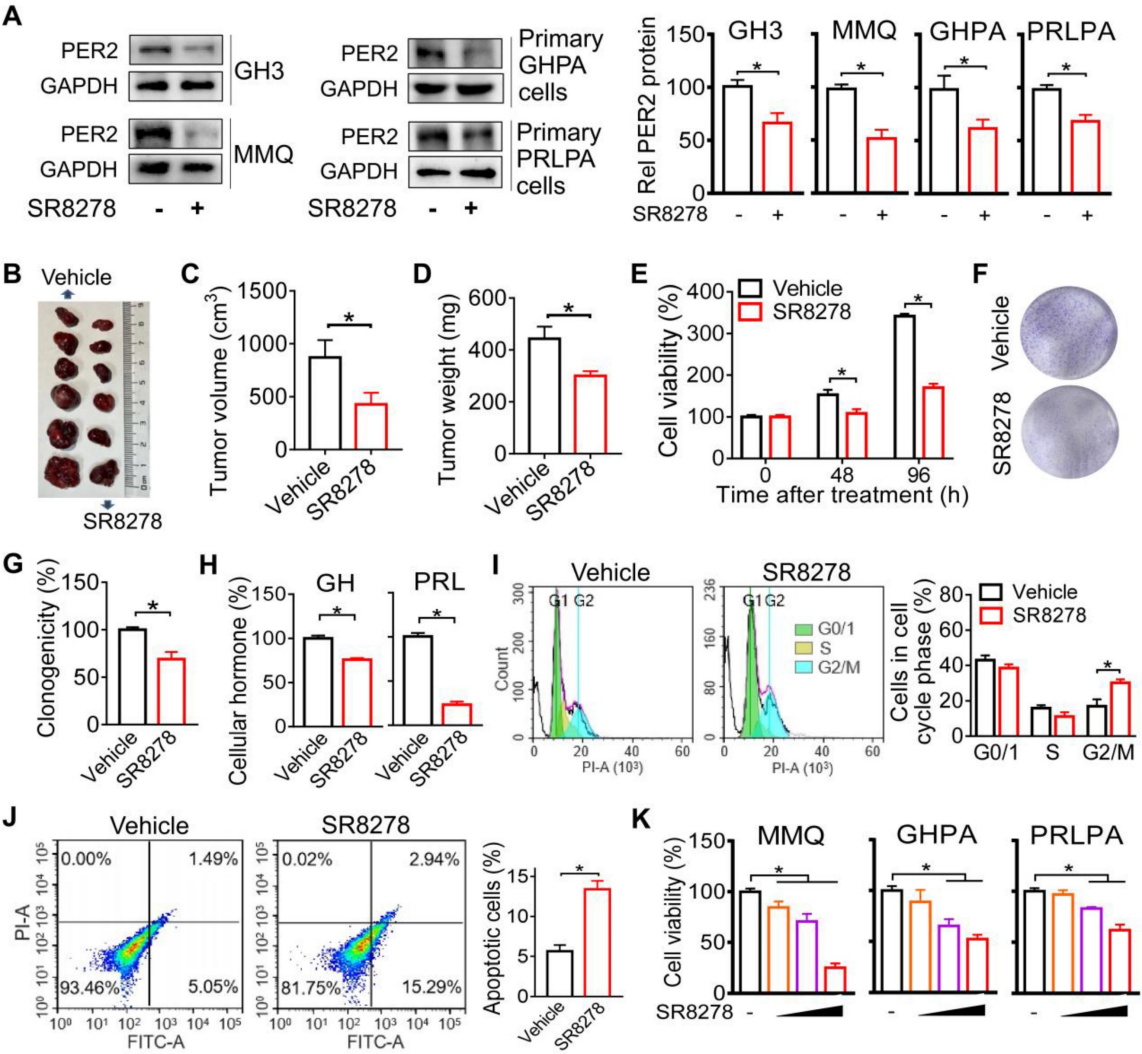
** Inhibition of PER2 limits pituitary tumorigenesis.** (A) Effects of SR8278 on PER2 protein in GH3, MMQ, primary GHPA and primary PRLPA cells. (B) Effects of SR8278 on growth of GH3 xenograft tumor. (C) Effects of SR8278 on the volume of GH3 xenograft tumor. (D) Effects of SR8278 on the weight of GH3 xenograft tumor. (E) Effects of SR8278 on the viability of GH3 cells. (F) Representative photographs of colony formation of GH3 cells treated with SR8278 or vehicle. (G) Effects of SR8278 on colony formation of GH3 cells. (H) Effects of SR8278 on GH and PRL levels in GH3 cells. (I) Effects of SR8278 on cell cycle phase distribution in GH3 cells. (J) Effects of SR8278 on apoptosis of GH3 cells. (K) Effects of SR8278 on viability of MMQ, primary GHPA and primary PRLPA cells. In panels A, E, G, H, I, J and K, data are mean ± SEM (*n* = 3 biologically independent samples). In panels C and D, data are mean ± SEM (*n* = 6 biologically independent samples). In panels A, C, D, E, G, H, I and J, *p < 0.05 (t-test). In panel K, *p < 0.05 (one-way ANOVA with Bonferroni post hoc test).

Given that PER2 promotes pituitary tumorigenesis, we wondered whether modulation of PER2 by a small molecule can affect pituitary tumorigenesis. We found that SR8278 (initially identified as an antagonist of REV-ERBα 27) can down-regulate the expression of PER2 in GH3, MMQ, primary GHPA and primary PRLPA cells (Figure 6A), and thus tested its effects on pituitary tumorigenesis. Down-regulation of PER2 by SR8278 may involve antagonism of REV-ERBα and up-regulation of its target NFIL3 that can directly repress expression of PER2 28,29. SR8278 (50 mg/kg) was intraperitoneally injected into GH3 xenograft tumor-bearing mice at ZT14 (corresponding to a peak PER2 expression) for 10 days. SR8278 treatment significantly inhibited the tumor growth in mice, as evidenced by lower tumor volume and weight (Figure 6B-D). Furthermore, SR8278 reduced the viability and colony formation of GH3 cells (Figure 6E-G), accompanied by decreased cellular GH and PRL levels (Figure 6H). Moreover, SR8278 arrested the cells at G2/M phase and enhanced the apoptosis of GH3 cells (Figure 6I-J). Similar inhibitory effects of SR8278 on cell viability were observed in MMQ, primary GHPA and primary PRLPA cells (Figure 6K). Altogether, inhibition of PER2 by SR8278 protected mice from developing pituitary adenoma probably through limiting cell cycle progression.

### PER2 promotes the transcription of *Ccnb2*, *Cdc20* and *Espl1* via an interaction with HIF-1α

**Figure 7:**
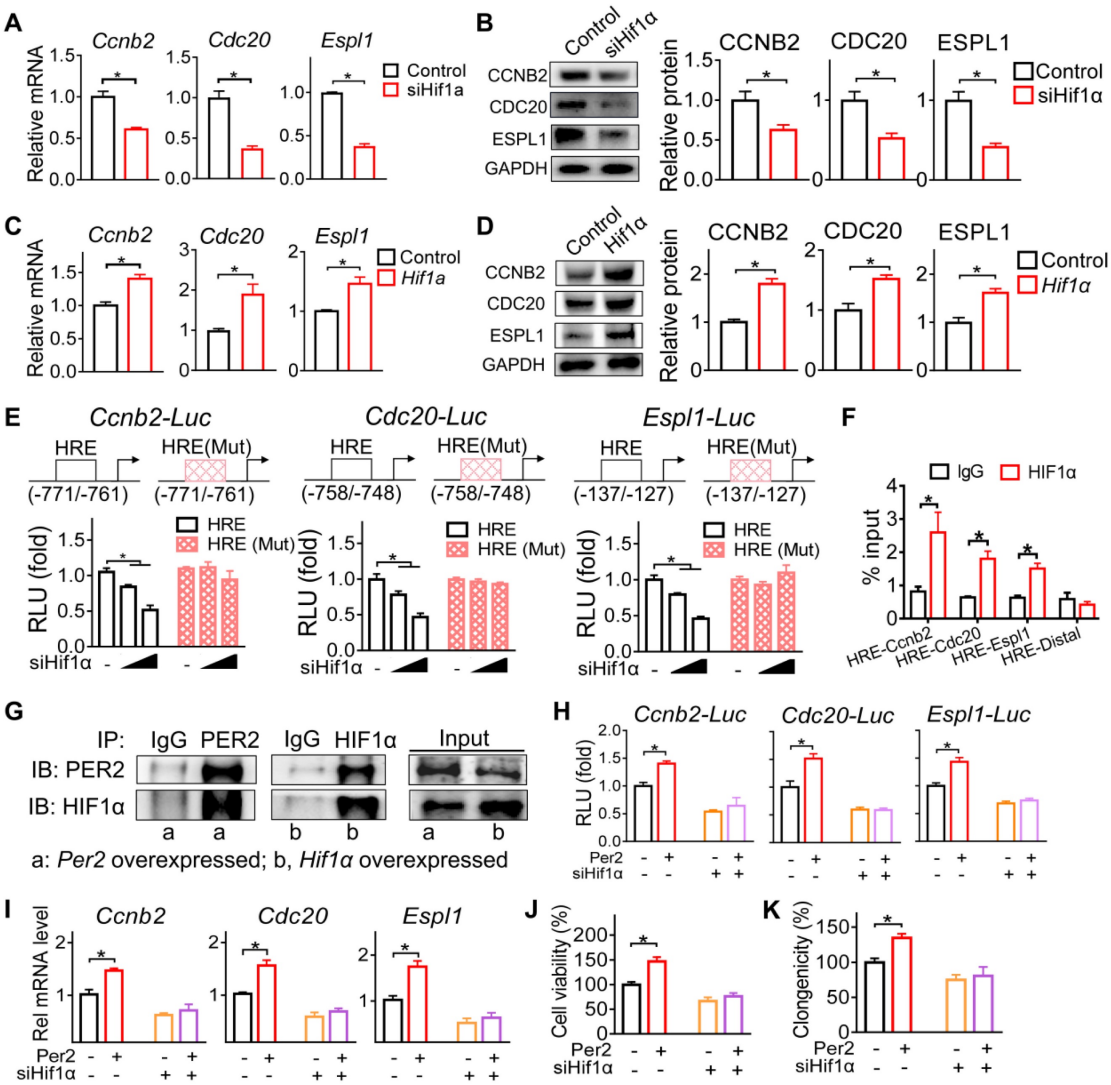
** PER2 promotes the transcription of *Ccnb2*, *Cdc20* and *Espl1* via an interaction with HIF-1α.** (A) Effects of *Hif-1α* knockdown on mRNA expression of *Ccnb2*, *Cdc20* and *Espl1* in GH3 cells*.* (B) Effects of *Hif-1α* knockdown on protein expression of CCNB2, CDC20 and ESPL1 in GH3 cells. (C) Effects of *Hif-1α* overexpression on mRNA expression of *Ccnb2*, *Cdc20* and *Espl1* in GH3 cells*.* (D) Effects of *Hif-1α* overexpression on protein expression of CCNB2, CDC20 and ESPL1 in GH3 cells. (E) Effects of *Hif-1α* on the activities of *Ccnb2*, *Cdc20* and *Espl1* luciferase reporters and their HRE-mutated versions*.* (F) ChIP-qPCR analysis of HIF-1α enrichment at promoter regions of *Ccnb2*, *Cdc20* and *Espl1* in GH3 tumor. (G) Co-IP results of PER2 and HIF-1α in GH3 cells. (H) Effects of pcDNA3.1-*Per2* and/or siHif-1α on the activities of *Ccnb2*, *Cdc20* and *Espl1* luciferase reporters. (I) Effects of pcDNA3.1-*Per2* and/or siHif-1α on mRNA levels of *Ccnb2*, *Cdc20* and *Espl1* in GH3 cells. (J) Effects of pcDNA3.1-*Per2* and/or siHif-1α on viability of GH3 cells. (K) Effects of pcDNA3.1-*Per2* and/or siHif-1α on colony formation of GH3 cells. Data are mean ± SEM (*n* = 4 biologically independent samples). In panels A, B, C, D, H, I, J and K, *p < 0.05 (t-test). In panel E, *p < 0.05 (two-way ANOVA with Bonferroni post hoc test).

Since PER2 generally acts as a repressor of gene transcription and expression 30, we reasoned that an intermediate regulator (as a mediator) is necessary for the positive regulation of *Ccnb2*, *Cdc20* and *Espl1* by PER2. A survey of the literature suggested HIF-1α as a candidate for such intermediate regulator because PER2 functions as an effector protein for the recruitment of HIF-1α to the specific response element (i.e., the hypoxia-response element, HRE) and thus promotes the transcription of its target genes 31. We therefore tested whether HIF-1α has a role in PER2 regulation of CCNB2, CDC20 and ESPL1. We found that *Hif-1α* knockdown (by a specific siRNA) decreased both mRNA and protein expression levels of CCNB2, CDC20 and ESPL1, whereas *Hif-1α* overexpression increased their expression in GH3 cells (Figure 7A-D). Furthermore, the specific siRNA targeting *Hif-1α* dose-dependently inhibited the promoter activities of *Ccnb2*, *Cdc20* and *Espl1* based on luciferase reporter assays (Figure 7E). Promoter analysis predicted a potential HRE element (-771/-761 bp for *Ccnb2, -*758/-748 bp for* Cdc20* and -137/-127 bp for *Espl1*) in each promoter of *Ccnb2*, *Cdc20* and *Espl1* (Figure 7E). Mutation experiments confirmed that the predicted HRE elements were indeed required for the transcriptional actions of HIF-1α on these three genes (Figure 7E). Supporting this, ChIP (chromatin immunoprecipitation) assays demonstrated direct interactions of HIF-1α protein with *Ccnb2-*HRE, *Cdc20-*HRE and *Espl1*-HRE (Figure 7F). Therefore, we concluded that HIF-1α trans-activated *Ccnb2*, *Cdc20* and *Espl1* via direct binding to a HRE element in their promoters.

Next, we examined whether HIF-1α mediates regulation of *Ccnb2*, *Cdc20* and *Espl1* by PER2. In line with its known role as an effector molecular of HIF-1α 31, PER2 had a protein-protein interaction with HIF-1α (Figure 7G). According to luciferase reporter assays, PER2 activated the promoter activities of *Ccnb2*, *Cdc20* and *Espl1*, however, these activation effects were attenuated by *Hif-1α* silencing (Figure 7H). Likewise, *Hif-1α* silencing blunted the induction effects of PER2 on *Ccnb2*, *Cdc20* and *Espl1* mRNAs in GH3 cells (Figure 7I). Moreover, silencing of *Hif-1α* abated the promoting effects of PRE2 on pituitary cell proliferation according to cell viability and colony formation assays (Figure 7J-K). Altogether, these findings indicated that PER2 promotes the transcription and expression of *Ccnb2*, *Cdc20* and *Espl1* via an interaction with HIF-1α.

## Discussion

The role of circadian clock in pituitary tumorigenesis remains enigmatic, although patients with pituitary tumors have been reported to suffer from disrupted circadian rhythms since 1980s. In this study, we have uncovered a causal role of circadian misalignment in tumor development of GHPA and PRLPA. Importantly, up-regulation of PER2, arising from circadian desynchrony, underlies an accelerated growth of GHPA and PRLPA. Up-regulated PER2 increases pituitary cell proliferation and tumorigenesis by promoting the expression of cell cycle genes (*Ccnb2*, *Cdc20* and *Espl1*) via enhancement of HIF-1α-mediated transactivation (Figure S5). Thus, we have established PER2 as an integrator of circadian clock with pituitary adenomas, providing new insights to the molecular mechanisms behind clock-controlled tumorigenesis. However, the mechanisms by which the clock genes such as *Per2* are disrupted in pituitary tumors remains unresolved.

Associations of cancer pathogenesis with circadian rhythm disruption and PER2 malfunction have been recognized for multiple cancer types 19. For instance, mice with genetic alterations in *Per2* are more susceptible to developing salivary gland hyperplasia, lymphoma, teratomas, liver, lung and ovarian cancers 19. Also, *Per2* mutation accelerates Apc^Min/+^ tumorigenesis 32. These findings suggest a tumor-suppressive function for PER2. Paradoxically, other studies show that* Per2*-null mice are not tumor-prone, and *Per2* and other clock genes may fuel tumorigenesis with a tumor-promoting function as similarly observed here for the role of PER2 in pituitary tumorigenesis 33. Apparently, the divergent roles of PER2 in tumorigenesis depend on the cancer cell type or status. As noted by Sulli *et al*., the dual function of clock components in cancer development could be due to tissue- and cell-specific mechanisms pertinent to the maintenance of circadian homeostasis 20.

The mechanisms for regulation of tumorigenesis by circadian clock components are multi-factorial, and involve modulation of cell cycle, apoptosis, DNA repair, cytotoxic immunity and cancer metabolism 34. In this study, unbiased transcriptomic analysis revealed that deregulation of cell cycle pathway contributed to suppressed pituitary tumorigenesis in *Per2*-null mice, which were validated by a series of subsequent *in vitro* and *in vivo* studies (Figures 4 & 5). We examined pituitary expression of major genes involved in DNA repair, cytotoxic immunity and glucose metabolism as well as their regulators in *Per2*-null versus in control mice. These genes were barely affected by* Per2* ablation (Figure S6). We also analyzed the expression of known genes contributing to formation and progression of pituitary adenomas [e.g., *Ghrhr* (growth hormone-releasing hormone receptor) and *Pttg* (pituitary tumor-transforming gene)], and did not find their associations with *Per2* (Figure S7). Thus, PER2 promotes tumor growth of GHPA and PRLPA primarily via accelerating cell cycle progression. It is noteworthy that aberrant epigenetic modifications (such as DNA methylation, RNA modification, and histone modifications) have been observed in various tumors including pituitary tumors 35,36. However, it remains to be determined whether epigenetic modifications are involved in PER2 regulation of pituitary tumors. In addition, immune cell infiltration plays a role in development and progression of various tumors including pituitary tumors 37,38. Whether immune cell infiltration has a role in PER2 regulation of pituitary tumors requires further investigations.

Our study suggests the core clock component PER2 as a novel common drug target for management of GHPA and PRLPA because PER2 acts as a pro-oncogenic protein for these two types of pituitary adenomas and the small molecule SR8278 decreases PER2 expression and mitigates tumor growth. In fact, targeting clock components such as REV-ERBs, RORs and CRYs for cancer prevention and treatment have been proposed for multiple cancer types 20. For instance, REV-ERB agonists (SR9009 and SR9011) show selective anticancer effects in leukemia, brain, breast, and colon cancer cells by inhibiting autophagy and de novo lipogenesis 39. Pharmacological inhibition of RORγ exhibits potent antitumor properties in pancreatic adenocarcinoma, prostate and breast cancers 19. It is noteworthy that the tumor-promoting effects of PER2 may be not limited to GHPA and PRLPA only because elevations in PER2 protein were similarly observed in other subtypes of pituitary adenomas such as TSH- and ATCH-secreting types (Figure S1C). Thus, it is likely that PER2 may have a broad role in pituitary tumorigenesis independent of cell types.

PER2 oscillates in normal pituitary gland in mice with peak expression in the early dark phase and trough expression in the early light phase (Figure S2). In pituitary adenomas, the rhythmicity of PER2 retained but with a decreased amplitude (Figure S8A & Figure S2). Diurnal rhythm of PER2 parallels those of cell cycle genes (*Ccnb2*, *Cdc20* and *Espl1*) in pituitary tumors, supporting PER2 as a driver of rhythmicity in these cell cycle genes and implicating a diurnal rhythm in cell cycle (Figure S8B). In fact, pituitary adenoma cell proliferation displays a robust 24-h rhythm with a higher rate of proliferation during the dark period (Figure 2G-I), consistent with the diurnal profile of PER2 as a positive cell cycle regulator. Cyclic expression of PER2 may impact the therapeutic potency of drugs targeting this protein with respect to the time of dosing. However, whether this is true or not requires case-by-case investigations, as other diurnal factors such as temporal pharmacokinetic parameters also contribute to drug chronoeffects 40.

In sum, we uncover a causal role of circadian misalignment in growth of GHPA and PRLPA. Importantly, PER2 up-regulation, arising from circadian desynchrony, underlies accelerated growth of GHPA and PRLPA. Mechanistically, PER2 increases pituitary cell proliferation by promoting the expression of cell cycle genes (*Ccnb2*, *Cdc20* and *Espl1*) via enhancement of HIF-1α-mediated transactivation. These findings establish PER2 as an integrator of circadian clock with pituitary adenomas, providing new insights to the molecular mechanisms behind clock-controlled tumorigenesis.

## Materials and Methods

### Materials

17β-estradiol was purchased from Aladdin (Shanghai, China). SR8278 was purchased from Tocris Bioscience (Ellisville, MO). ELISA kits for mouse GH and PRL were obtained from Elabscience Biotechnology (Wuhan, China). Anti-GAPDH antibody was purchased from Abcam (Cambridge, MA), and anti-PER2 antibody from Affinity BioReagents (Golden, CA). Anti-HIF-1α antibody was purchased from Genetex (San Antonio, TX), and anti-CCNB2 antibody from Bioworld (Visalia, CA). Anti-CDC20 and anti-ESPL1 antibodies were obtained from Bioss Biotechnology (Woburn, MA). pcDNA3.1, pcDNA3.1-*Per2,* pRL-TK and luciferase reporters of *Ccnb2* (-2000/100 bp),* Cdc20* (-2000/100 bp) and *Espl1* (-2000/100 bp) were obtained from Transheep Technologies (Shanghai, China). siPer2 (siRNA targeting *Per2*, sequence is shown in Table S2) was synthesized by IGE Biotechnology (Guangzhou, China). Adenoviral vector encoding siPer2 was obtained from HanBio Biotechnology (Shanghai, China).

### Animals

*Per2* knockout mice (*Per2*^-/-^ mice, on a C57BL/6 background) have been described in our previous report 30. BALB/c-nude mice (5-6 weeks old) and Wistar-Furth rats (5-7 weeks old) were obtained from SiPeiFu Biotechnology (Beijing, China). All animals, receiving food and water ad libitum, were kept under a 12 h light/dark cycle [light on at 6:00 AM (= ZT0) and light off at 6:00 PM (= ZT12)]. All animal studies were approved by the Institutional Animal Care and Use Committee of Guangzhou University of Chinese Medicine.

### Xenograft experiments

A xenograft tumor model of pituitary adenoma in nude mice was established as described previously 41. In brief, 3×10^6^ normal GH3 cells or adenovirus (Ad.siPer2 or control) transfected GH3 cells were suspended in serum-free DMEM (Dulbecco's Modified Eagle Medium), mixed with isopyknic matrigel matrix (Corning Corp, Bebford, MA), and injected subcutaneously into the right posterior back of BALB/c nude mice. Tumor growth was recorded every other day for 16 days. Tumor volume was calculated as length × width^2^× 1/2. After sacrifice of mice, tumors were excised, weighed, and subjected to immunofluorescence and RT-qPCR (real-time quantitative polymerase chain reaction). Plasma samples were collected for measurements of GH and PRL. To assess the effects of PER2 inhibition on tumor growth, nude mice were injected with GH3 cells. After one week, we initiated the treatment with SR8278 (50 mg/kg) or vehicle via intraperitoneal injection for 10 days. Tumor growth was recorded and samples were collected as described above.

### Jetlagged models

Jetlagged mice were established according to a published method 42. In brief, nude mice were subjected to a jetlag schedule of 8 h advance in light/dark cycle every 2 days for 20 days. We initiated subcutaneous injection of GH3 cells in the fifth day. The mice continued on the light advancing schedule for another 15 days. Control nude mice were kept under a standard 12 h light/dark cycle. Tumor growth was recorded and samples were collected as described above.

### Estrogen-induced PRLPA

*Per2*^-/-^ mice and wild-type littermates (8-12 weeks old) were subcutaneously implanted with Alzet osmotic pumps (model 2004, Durect Corporation, Cupertino, CA) containing 20 mg 17β-estradiol to induce PRLPA according to the manufacturer's protocol. Three months later, mice were sacrificed to collect plasma and tumor samples for further analysis.

### Human specimens

Surgical pituitary samples were collected at 9:00 AM~12:00 PM from patients with GHPA, PRLPA, ATCHPA, TSHPA or NFPA from the First Affiliated Hospital of Jinan University. Normal pituitary samples were autopsy specimens from individuals who died (at 9:00 AM~12:00 PM) from accidental causes.

### Isolation and culture of primary pituitary cells

Isolation of primary pituitary cells were performed as previously described 43. In brief, human pituitary adenoma and rat normal pituitary tissues were minced and incubated with 0.5% collagenase type I and 0.05% DNase I (Solarbio, Beijing, China) at 37℃ for 45 min. The resulting mixture was centrifuged and the supernatant was collected. Then the tissues were incubated once more with 0.25% trypsin at 37℃ for 15 min, followed by centrifugation. Supernatant was collected, pooled with the former supernatant and flushed over a 70 μm filter. The filtrate was centrifuged and the pellets (pituitary cells) were collected. Cells were then cultured in DMEM medium (Life technologies, Waltham, MA) with 10% fetal bovine serum (FBS) (Gibco, New York) and 1% penicillin/streptomycin (Gibco, New York).

### Cell viability

Cell Counting Kit-8 (CCK-8, Biosharp Biotech, Beijing, China) was used to assess the cell viability. GH3 and MMQ cells were maintained in Ham's F-12K medium (Procell Biotech, Wuhan, China) supplemented with 2.5% FBS, 15% horse serum, and 1% penicillin/streptomycin. Cells were transfected with overexpression plasmid (pcDNA3.1-*Per2* or pcDNA3.1-*Hif-1α*) or siRNA (siPer2 or siHif-1α) or control using jetPRIME transfection reagent (Polyplus, Illkirch, France). After transfection for 24, 48, 72 and/or 96 h, cells were incubated with CCK-8 stock solution for 2 h. The number of viable cells was determined at 450 nm using a Synergy HT Multi-Mode Microplate Reader (BioTek, Winooski, VT). In another set of experiments, cells were treated with SR8278 or vehicle for 48 and/or 96 h. Cell viability was assayed as described above.

### Clonogenic assay

Cells were fixed in 4% paraformaldehyde and stained with 0.1% crystal violet (Acmec, Shanghai, China) for 15 min at room temperature. Cell plates were photographed after washing and drying. The colonies were counted by using Image-Pro Plus 6.0.

### Wound healing assay

GH3 cells were seeded into six-well plates and transfected with siPer2 or control using jetPRIME for 48 h. Wounds were made in the cell monolayer by making scratches with a 10 μl pipette tip. After 24 h, cell plates were washed to remove non-adherent cells and photographed using an inverted microscope.

### Flow cytometry

Flow cytometry was performed as previously described 44. For cell cycle analysis, cell samples were stained with propidium iodide (Invitrogen, Auckland, New Zealand) for 30 min at room temperature. For apoptosis assay, cells were co-incubated with a fluorescein isothiocyanate-labeled annexin V antibody (Invitrogen, Auckland, New Zealand) and propidium iodide for 10 min. The cell cycle phase distribution and apoptosis were detected using ACEA NovoExpress software (ACEA Biosciences, San Diego, CA).

### Locomotor activity analysis

Mice were housed in individual cages equipped with running wheels (Lafayette Instrument, Lafayette, IN), and were placed in light-tight cabinets under a 12 h light/dark cycle. After acclimation to the system, mice were subjected to continuous recording for 7 days. The locomotor activity of mice was analyzed using the ClockLab sftware (Actimetrics, Wilmette, IL).

### EEG and EMG recordings

EEG (electroencephalogram) and EMG (electromyography) recordings were performed as described in our recent report 29. Mice were anesthetized and mounted in a stereotaxic apparatus. Screw electrodes were inserted into mouse skull (coordinates: +2 mm bregma, ±1 mm midline for two recording electrodes; -2 mm Bregma, -1 mm midline for a reference electrode, and +1 mm midline for a ground electrode) to measure cortical EEG. Stainless steel electrodes were implanted in dorsal neck muscle to measure EMG. After recovery for 7 days, mice were subjected to EEG and EMG recordings. Data were acquired using a tethered data acquisition system (Medusa, Biosignal technologies, Nangjing, China) with a resolution of 500 Hz. Waveforms were visualized using Sirenia Sleep Pro software (Pinnacle technologies, Lawrence, KS). EEG signals were high-pass filtered (> 0.5 Hz) and EMG was band-pass filtered between 5 to 45 Hz. These data were used to define the vigilance states of wake, NREM (non-rapid eye movement) sleep, and REM (rapid eye movement) sleep by an automatic script (Lunion Stagesoftware, LunionData, Nangjing, China).

### LC-MS/MS

Corticosterone and serotonin were quantified using an LC-MS/MS (liquid chromatography-tandem mass spectrometry) system (LCMS-8050, Shimadzu, Kyoto, Japan) and a C18 column (Phenomenex, Torrance, CA). The mobile phases were acetonitrile (mobile phase A) and water (mobile phase B) containing 0.1% formic acid. The gradient elution program was: 0-2 min, 98% B; 2-3 min, 98-10% B; 3-5 min, 10% B; 5-7min, 10-98% B. Detection was carried out in a positive ion mode. The column flow rate was set at 0.3 ml/min. The mass transition ion pairs were selected as *m/z* 347.3 → 121.15 for corticosterone, and *m/z* 176.95 → 159.95 for serotonin.

### RNA sequencing

Pituitary gland samples were collected from *Per2^-/-^* mice and wild-type littermates at ZT14. RNA was isolated using Trizol (Takara, Otsu, Japan) according to the manufacturer's instructions. RNA was quantified using Qubit 2.0 Fluorometer (Life Technologies, Carlsbad, CA) and the quality was checked using Bioanalyzer 2100 RNA 6000 Nano Kit (Agilent Technologies, Santa Clara, CA). RNA samples were considered qualified when RIN > 7.7. RNA-sequencing was performed as described in our previous report 45. Differentially expressed genes were defined when FPKM > 0.1, |fold change| > 2 and false discovery rate < 0.05. Furthermore, KEGG and GO analyses were performed based on differentially expressed genes (DEGs) as previously described 46.

### RT-qPCR and Western blotting

Experimental procedures of RT-qPCR and Western blotting have been described in our previous publication 47. Primers used in RT-qPCR are listed in Table S2.

### Luciferase reporter assay

GH3 cells were co-transfected with a luciferase reporter (*Ccnb2-Luc, Cdc20-Luc* or* Espl1-Luc* or a mutated version), pRL-TK, and siHif-1α using jetPRIME in the presence or absence of pcDNA3.1-*Per2*. After 36 h, cells were lysed in passive lysis buffer. Luciferase activities were assayed using the Dual-Luciferase Reporter Assay System (Promega, Madison, WI) and GloMax 20/20 luminometer (Promega, Madison, WI). Firefly luciferase activity was normalized to renilla luciferase activity and expressed as a relative luciferase unit.

### ChIP

ChIP assay was performed using a SimpleChIP Enzymatic Chromatin IP Kit (Cell Signaling Technology, Beverly, MA) as described in our previous report 47. In brief, GH3 tumors were fixed in 37% formaldehyde and digested with micrococcal nuclease. Sheared chromatin was immunoprecipitated with anti-HIF-1α (Genetex, San Antonio, TX) or normal rabbit IgG (control) at 4℃ overnight. Purified DNAs were analyzed by qPCR with specific primers (Table S2).

### Co-IP

Co-IP (co-inmunoprecipitation) was performed as described in our previous report 48. In brief, GH3 cells were transfected with pcDNA3.1-*Per2* or pcDNA3.1-*Hif-1α* for 48 h, and lysed in lysis buffer (Beyotime, Shanghai, China). Lysate was incubated with anti-PER2 (Affinity, Golden, CA), anti-HIF-1α (Genetex, San Antonio, TX) or anti-normal rabbit IgG (CST, Beverly, MA) overnight, followed by incubation with protein A/G magarose beads (EpiZyme Biotech, Shanghai, China) for 4 h. Bound proteins were analyzed by Western blotting.

### H&E staining

Pituitary tissues were fixed in 4% paraformaldehyde and embedded in paraffin. 4 µm thick sections were prepared for H&E (haematoxylin and eosin) staining as previously described 45. Histopathological changes were evaluated based on the degree of vacuolation, vascular lakes, nuclear pleomorphism and mitosis according to a previous report 49. At least three regions from each section and three sections were imaged for each animal.

### Immunofluorescence

20 µm thick coronal sections were prepared from GH3 tumor and pituitary gland tissues, permeabilized with 0.5% Triton X-100, blocked with 10% horse serum, and incubated with the primary antibodies against Ki67 (Affinity BioReagents, Golden, CA), phospho-histone H3 (Proteintech, Tokyo, Japan) and cleaved caspase-3 (Servicebio, Wuhan, China). Then the sections were incubated with DAPI (4',6-diamidino-2-phenylindole). Sections were imaged using a Nikon Optiphot fuorescent microscope (Tokyo, Japan). Ki67-, phospho-histone H3- and DAPI-positive cells were counted using Image-J software.

### Statistical analysis

Data are recorded as mean ± standard errors of the mean (SEM). Student's t-test was used to analyze a difference between the means of two groups. One-way or two-way ANOVA followed by Bonferroni post hoc test was used for multiple group comparisons. All statistical analyses were performed with GraphPad Prism 7.0 (GraphPad Software, San Diego, CA). The level of significance was set at *p* < 0.05 (*).

## Supplementary Material

Supplementary figures and tables.

## Abbreviations

ACTHPA: adrenocorticotropic hormone-secreting pituitary adenoma
Bak1: BRI1-associated kinase 1
Bcl2: B-cell lymphoma 2
Bid: BH3-interacting domain death agonist
BMAL1: brain and muscle Arnt-like protein 1
Ccna1: cyclin A1
Ccnb2: cyclin-B2
Ccnd1: cyclin-D1
Cdc20: Cell division cycle 20
Cdkn2a: cyclin dependent kinase inhibitor 2A
ChIP: chromatin immunoprecipitation
CLOCK: circadian locomoter output cycles protein kaput
c-Myc: cellular-myelocytomatosis oncogene
Co-IP: co-inmunoprecipitation
Cry: cryptochrome
Dbp: D site-binding protein
DEGs: differentially expressed genes
E2f1: E2F transcription factor 1
EEG: electroencephalogram
EMG: electromyography
Espl1: extra spindle pole bodies like 1
GH: growth hormone
GHPA: growth hormone (GH)-secreting pituitary adenoma
Ghrhr: growth hormone-releasing hormone receptor
HIF-1α: hypoxia-inducible factor 1α
HRE: hypoxia-response element
HTLV-1: human T-cell leukemia virus 1
IARC: international agency for research on cancer
NFIL3: nuclear factor interleukin-3
NFPA: nonfunctioning pituitary adenoma
Nr1d1: nuclear receptor subfamily 1 group D member 1
PER2: period 2
PRL: prolactin
PRLPA: prolactin (PRL)-secreting pituitary adenoma
Pttg: pituitary tumor-transforming gene
Ror: retinoic acid-related orphan receptor
RT-qPCR: real-time quantitative polymerase chain reaction
TLK2: tousled like kinase 2
TSHPA: thyroid-stimulating hormone-secreting pituitary adenoma
LC-MS/MS: liquid chromatography-tandem mass spectrometry
S. pombe): Wee1, WEE 1 homolog 1
ZT: zeitgeber time
