# Supplementary material for: PER2 integrates circadian disruption and pituitary tumorigenesis
Source: Theranostics. 2023 Apr 29;13(8):2657–72. doi: 10.7150/thno.82995 (PMC10196825; doi:10.7150/thno.82995)
Supplement: Supplementary file 1 — Supplementary figures and tables. [file thnov13p2657s1.pdf]

Supplemental Materials

Manuscript title:

PER2 integrates circadian disruption and pituitary tumorigenesis

Table S1. Differentially expressed genes in the top ten KEGG pathways.

	**KEGG pathways**	**Genes**
1	Cell cycle	*Ccnb2, Cdkn2a, E2f1, Ccna1, Cdc25c, Cdc20, Espl1*
2	Type II diabetes mellitus	*Hk3, Irs3, Cacna1e, Cacna1g*
3	Human T-cell leukemia virus 1 infection	*Ccnb2, Cdkn2a, E2f1, Il2rg, Ccna1, Cdc20, Espl1, H2-M10.2*
4	Neuroactive ligand-receptor interaction	*Fshb, C3ar1, Rxfp2, Avp, C5ar1, Ptafr, Oxtr, Gabrb2, Gpr35*
5	Base excision repair	*Pole, Neil3, Mid1*
6	Tuberculosis	*Tlr1, Clec7a, Fcgr2b, Coro1a, Cck, Tlr9*
7	Primary immunodeficiency	*Tnfrsf13b, Ptprc, Il2rg*
8	Cellular senescence	*Ccnb2, Cdkn2a, E2f1, Ccna1, Hipk2, H2-M10.2*
9	Homologous recombination	*Eme1, Rad54b, Bard1*
10	Measles	*Oas3, Tlr9, Fcgr2b, Oas1g, Il2rg*

Table S2. Oligonucleotides used in this study.

	Forward (5′to 3′)	Reverse (5′to 3′)
**siRNA**		
siPer2	GGCAUUACCUCCGAGUAUATT	UAUACUCGGAGGUAAUGCCTT
siHif-1α	GACUCAAGCAACUGUUAUA	UAUAACAGUUGCUUGAGUC
Control	UUCUCCGAACGUGUCACGUTT	ACGUGACACGUUCGGAGAATT
**RT-qPCR**		
hBMAL1	GCGCTAAAGGAGAGCTGACA	CTCGGTTGCTGAGAGGACAG
hCCNB2	CCGACGGTGTCCAGTGATTT	TGTTGTTTTGGTGGGTTGAACT
hCDC20	GCACAGTTCGCGTTCGAGA	CTGGATTTGCCAGGAGTTCGG
hCLOCK	TGGGAATCCCTCAACTCAAC	GACTGAGGGAAGGTGCTCTG
hCRY1	TTGGAAAGGAACGAGACGCAG	CGGTTGTCCACCATTGAGTT
hCRY2	AACCACGACGAGACCTACG	GAGTTGGCGTTCATTCGGG
hESPL1	CAGGCACTTATCCCGAGGTG	ACCCGAACCCAGAAAGTGAC
hGAPDH	CATGAGAAGTATGACAACAGCCT	AGTCCTTCCACGATACCAAAGT
hNFIL3	CGCCGGGACATTTTAATCGC	TGGGCCTCCTTCGTTATCTTG
hNR1D1	CCAACAACAACACAGGTGGCG	GGGGATGGTGGGAAGTAGGT
hPER1	ACGGGCCGAATCGTCTACA	TGGAACCATAGAAGACTCCCAC
hPER2	CTTCAGCGATGCCAAGTTTGT	CGGATTTCATTCTCGTGGCTTT
hRORa	CACGACGACCTCAGTAACTACA	TGGTGAACGAACAGTAGGGAA
mAip	GCGGATCTCATCGCAAGACTT	GTGGCCTTAGTGCCATCCTG
mAtm	CAGGAAACCCTGCTGACCAT	CTTCCTCCACGCCTTTCAGT
mAtr	CCAAAAGGAGGTAAGGTCAACA	CGGCTCGTGTGTATGCTTTG
mBrca1	TCTCTTGGGGCTTCTCCGT	ACTTCTTGAATTTGGACGGCA
mCcna1	GATACCTGCTCGGGGAAAGAG	GCATTGGGGAAACTGTGTTGA
mCcnb2	GCCAAGAGCCATGTGACTATC	CAGAGCTGGTACTTTGGTGTTC
mCdc20	TTCGTGTTCGAGAGCGATTTG	ACCTTGGAACTAGATTTGCCAG
mCdkn2a	CGCAGGTTCTTGGTCACTGT	TGTTCACGAAAGCCAGAGCG
mChek2	AGAAATAAAGTGGTGCGTGTGG	TCAGTTTCCACACTGGGAGC
mCreb	TGTACCACCGGTATCCATGC	AGGATTTCCCTTCGTTTTTGGG
mCtla4	CCCGAGTCTGTGTGGGTTC	ACCACTGAAGGTTGGGTCAC
mCxcl1	TGGCTGGGATTCACCTCAAG	CCGTTACTTGGGGACACCTT
mE2f1	GAGAAGTCACGCTATGAAACCTC	CCCAGTTCAGGTCAACGACAC
mEgfr	GCCACTACATTGATGGCCC	CTGCCATTGAACGTACCCAGA
mErbb2	CTGGCATTTTTGCCGGAGAG	GGAGAATCCGTCCCCGAATG
mEspl1	TCATCCTACTTCGCAATGGTTC	CTCTGCTCCCTTCCAAAACAG
mGhrh	GCAGAACCTCAATCGGAGAG	CATCCTGAAGGGAGGTGAGG
mGhrhr	CGGCTTTCCAAGTCAACACTTC	AGCAGTAGAGGACAGCAACA
mGnas	TGGAGGAGAGGCGCAAAC	TCTCACTATCTCCGTTAAACCC
mIfng	ACTGCATCTTGGCTTTGCAG	ACCATCCTTTTGCCAGTTCCT
mIL-10	GGTGAGAAGCTGAAGACCCT	TCCAGCAGACTCAATACACACT

	Forward (5′to 3′)	Reverse (5′to 3′)
mMen1	CGCTAGGGAACTTGGCAGAC	ATCCTCCCGGCAGTAGTTGT
mPer2	AAAGCTGACGCACACAAAGAA	ACTCCTCATTAGCCTTCACCT
mPomc	CGTCCAAACCCTCGTTTCTCT	GCACCAGCTCCACACATCTAT
mPpar-y	CGGGCTGAGAAGTCACGTT	CATCACGGAGAGGTCCACAG
mPrlr	GACTCAAGGGGGCAAAGTCA	CACCTCCACAGAGAAGCGTT
mPttg	CGTTGGTGGCGCAGTCTT	CCTTTCTGCTGGCTTTAGGC
mRb1	TTTGTCCTTCCCGTGGATTCT	CCTTCTCCATCCTTGGACTGC
mRunx2	CCATCCATCCACTCCACCAC	TGCCTGGGGTCTGAAAAAGG
mTrp53	CGTGCTCACCCTGGCTAAAG	ATCCGACTGTGACTCCTCCA
mXpa	ACCACTTTGATCTGCCAACG	CTGTGAATGGCGTGGGTTCT
mβ-actin	GTGACGTTGACATCCGTAAAGA	GCCGGACTCATCGTACTCC
rBmal1	TGCCACTGACTACCAAGAAAGT	ATTTTGTCCCGACGCCTCTT
rCcnb2	CTGCTTCCTGCCTCTCTCAG	GCCATGTGCTGCATGACTTC
rCdc20	CGTGTTCGAGAGCGATTTGC	CTCCAGGTTTGCTAGGGGTG
rEspl1	CGGCCTGGAGGGTCTGG	CCTGTCTTCTCTCAGCATCGG
rMki67	ACAGGGCTTAGGAAACAGTCC	GGGTTCTAACTGGTCTTCCTGG
rPer2	TGCGAAGCGCCTCATTC	GCTGCTCATGTCCACGTCTT
rβ-actin	TACAACCTTCTTGCAGCTCCTC	CTTCTGACCCATACCCACCA
**ChIP**		
Ccnb2-HER	GGCACGCCTTAAATTCCACC	GAGCAACGCCCATTTGGTTT
Cdc20-HER	TCCTTGCATTGGGCCTTAGT	CGGTGGAATTTAAGGCGTGC
Espl1-HER	GCAGGGTGACTGTAGTTGAC	GAGTTGTAGTTTACCCACCGC
Distal	TGCACACTAGGCATCTGCTTTA	GCACCAGACACATAGGGTCAC

h, human; m, mouse; r, rat.

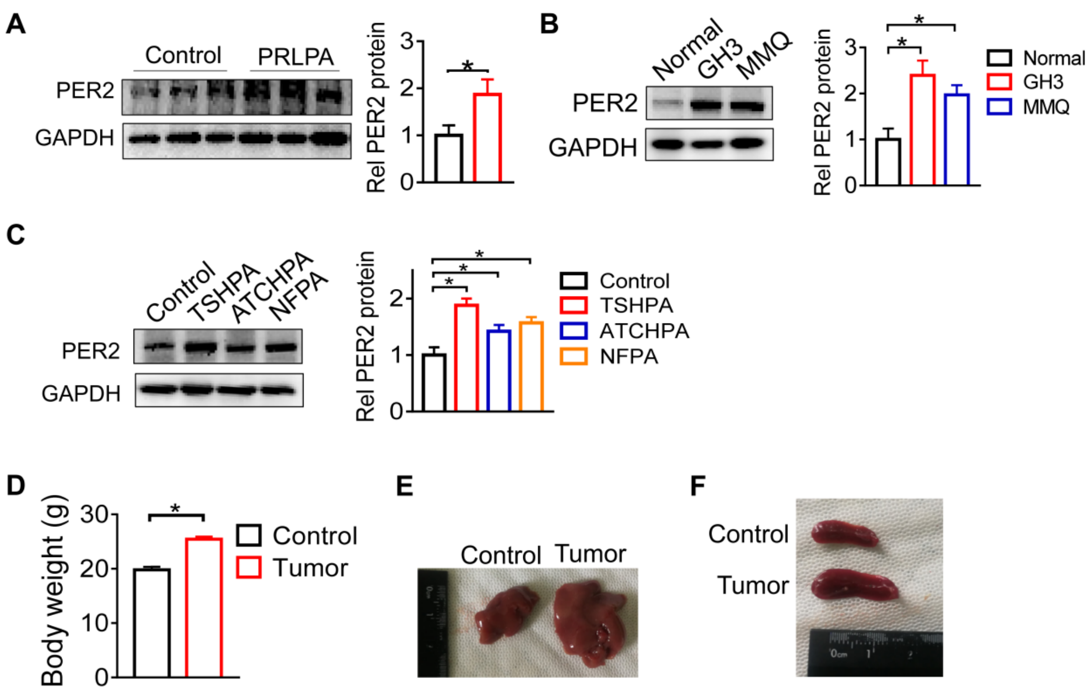

Figure S1. Disruption of pituitary clock genes in patients and animals with pituitary adenoma. (**A**) Pituitary PER2 is up-regulated in a mouse model of PRLPA induced by estrogen. (**B**) PER2 protein in rat normal pituitary cells as well as in GH3 and MMQ cells. (**C**) Relative pituitary PER2 protein levels in patients with TSHPA (thyroid-stimulating hormone-secreting pituitary adenoma), ACTHPA (adrenocorticotropic hormone-secreting pituitary adenoma) and NFPA (nonfunctioning pituitary adenoma) and in control indiviaduals. (**D**) Body weight of GH3 xenograft tumor-bearing and control mice. (**E**) A comparison of livers from GH3 xenograft tumor-bearing and control mice. (**F**) A comparison of spleen tissues from GH3 xenograft tumor-bearing and control mice. In panels A, B and C, data are mean ± SEM (n = 3 biologically independent samples). *$p < 0.05$ (t-test). In panel D, data are mean ± SEM ($n = 8$ biologically independent samples). *$p < 0.05$ (t-test).

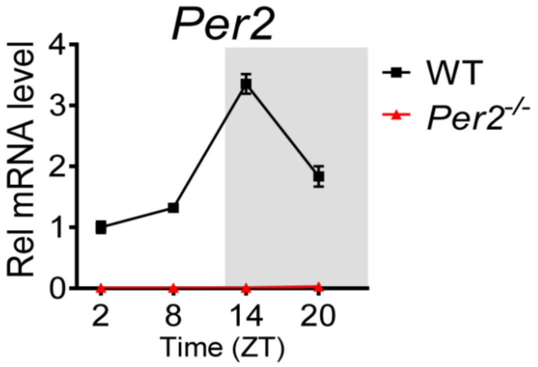

Figure S2. Diurnal mRNA expression of pituitary *Per2* in wild-type (WT) and Per2 knockout (*Per2*$^{-/-}$) mice. Data are mean ± SEM (*n* = 3 biologically independent samples).

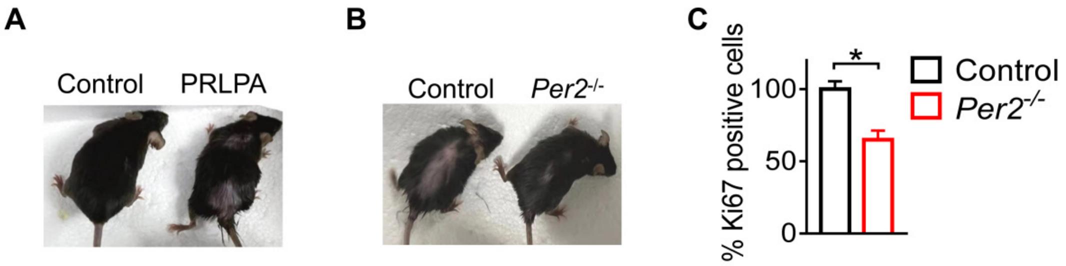

Figure S3. *Per2* ablation in mice restrains pituitary tumorigenesis. (**A**) A comparison of hair loss in control mice and mice with estrogen-induced PRLPA. (**B**) Hair loss in estrogen-treated *Per2*$^{-/-}$ and control mice. (**C**) A comparison of Ki67 levels in estrogen-treated *Per2*$^{-/-}$ and control mice. Data are mean ± SEM (n = 6 biologically independent samples). *$p < 0.05$ (t-test).

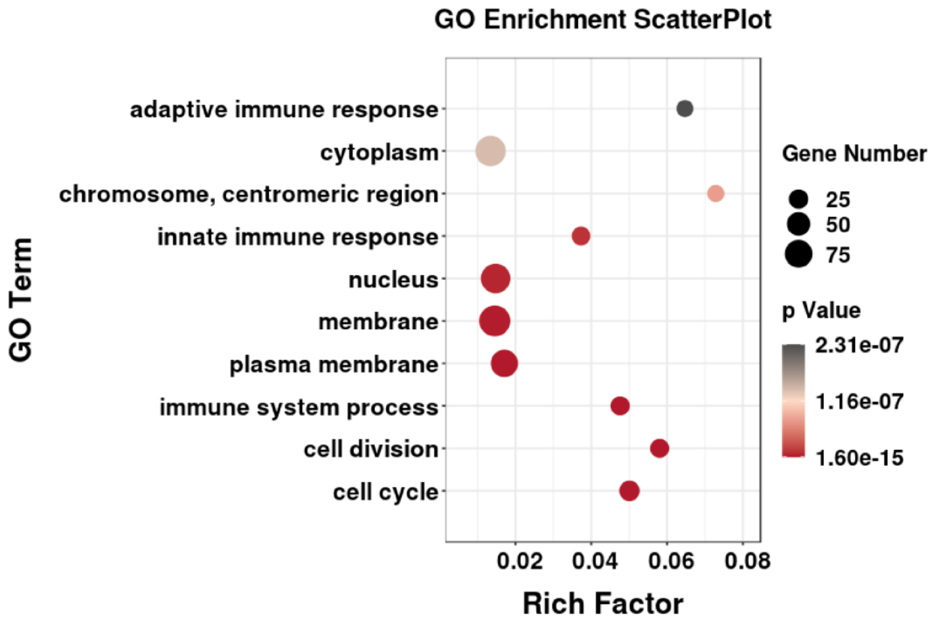

Figure S4. Gene Ontology (GO) analysis for differentially expressed genes in pituitary glands from estrogen-treated *Per2*$^{-/-}$ and wild-type mice.

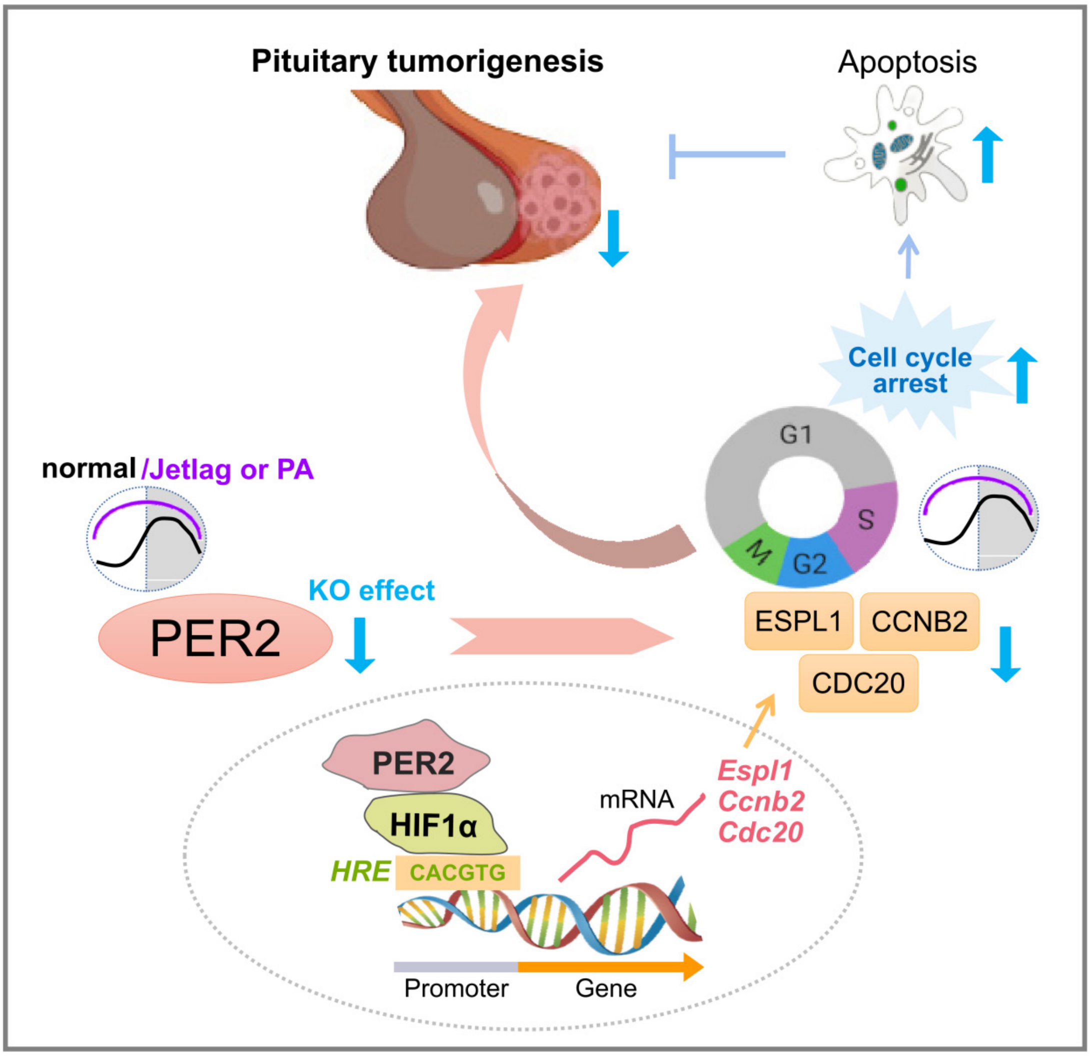

Figure S5. Schematic diagram showing the mechanism for integration of circadian disruption and pituitary tumorigenesis by PER2. Up-regulated PER2 due to circadian disruption increases pituitary cell proliferation and tumorigenesis by promoting the expression of cell cycle genes (*Ccnb2*, *Cdc20* and *Espl1*) via enhancement of HIF-1α-mediated transactivation. PA, pituitary adenoma.

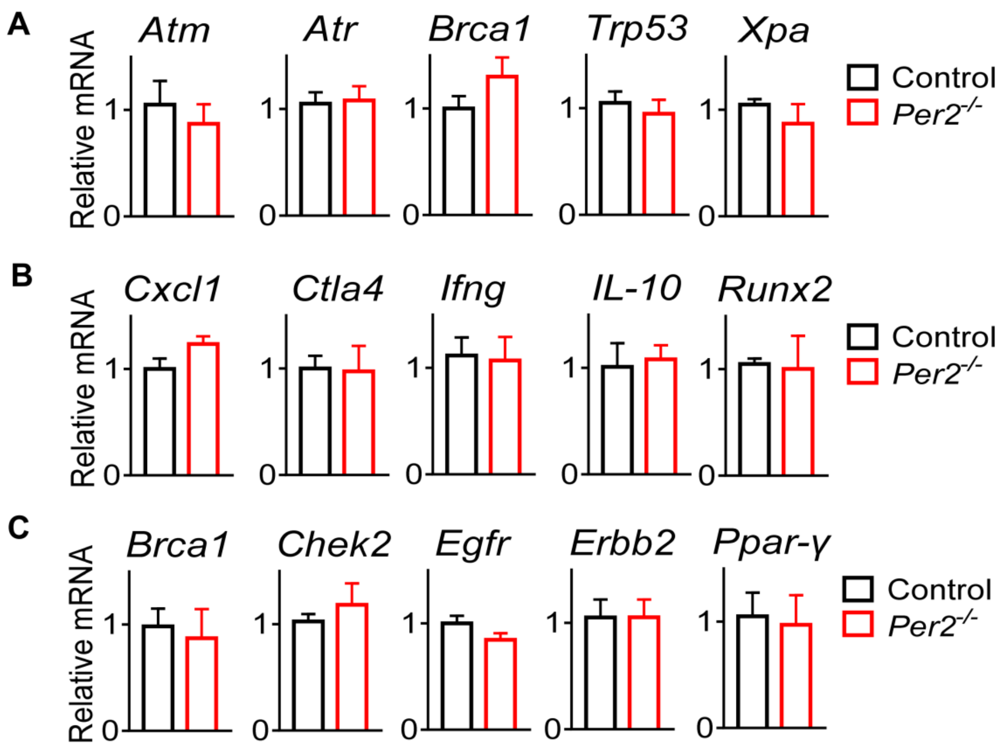

Figure S6. Pituitary mRNA expression of genes involved in DNA repair (A), cytotoxic immunity (B) and glucose metabolism (C) in *Per2*$^{-/-}$ and wild-type mice. Data are mean ± SEM (*n* = 3 biologically independent samples).

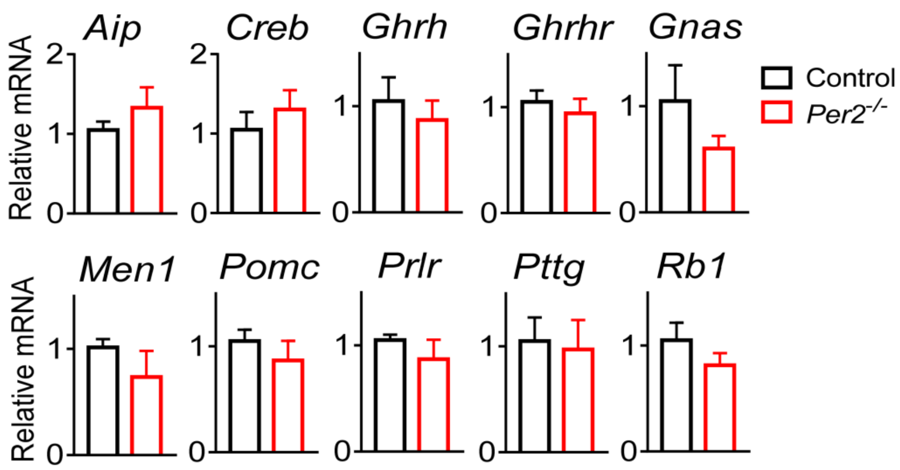

Figure S7. Pituitary mRNA expression of genes contributing to formation and progression of pituitary adenomas in *Per2*$^{-/-}$ and wild-type mice. Data are mean ± SEM (n = 3 biologically independent samples).

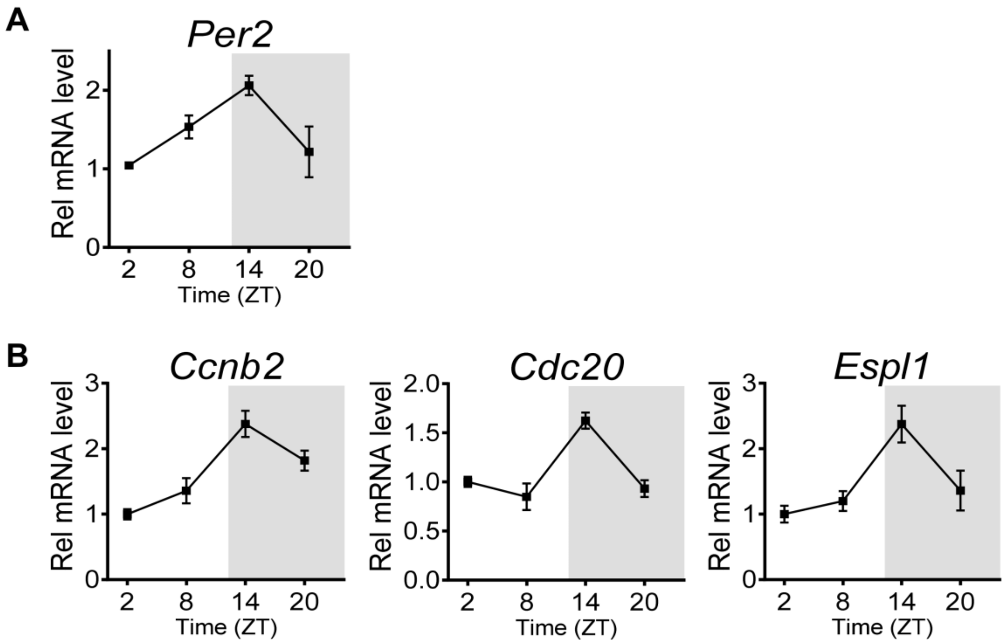

Figure S8. **Diurnal profile of *PER2* parallels those of *Ccnb2*, *Cdc20* and *Espl1* in pituitary tumors.** (**A**) Diurnal mRNA expression of pituitary *Per2* in mice with estrogen-induced PRLPA. (**B**) Diurnal mRNA expression of pituitary *Ccnb2*, *Cdc20* and *Espl1* in mice with estrogen-induced PRLPA. Data are mean ± SEM (n = 3 biologically independent samples).
